# The Bed Nucleus of the Stria Terminalis—Paraventricular Nucleus of the Hypothalamus Neural Circuit Regulates Neuropathic Pain Through the Brain-Spleen Axis

**DOI:** 10.1007/s12264-025-01454-9

**Published:** 2025-08-20

**Authors:** Shoumeng Han, Xin Chen, Li Ma, Xin Zeng, Ying Wang, Tingting Xie, Fancan Wu, Kun Song, Kenji Hashimoto, Hanbing Wang, Long Wang

**Affiliations:** 1 Department of Anesthesiology, Renmin Hospital of Wuhan University, Wuhan, 430060 China; 2 Department of Anesthesiology, Shaanxi Provincial People’s Hospital, Xi’an, 710068 China; 3 Department of Anesthesiology, The First People’s Hospital of Foshan, Foshan, 528000 China; 4 Chiba University Center for Forensic Mental Health, Chiba, 260-8670 Japan

**Keywords:** Brain-Spleen axis, Neuropathic pain, Splenectomy, Splenic denervation, Splenic sympathectomy

## Abstract

Neuropathic pain is a chronic condition caused by damage or dysfunction in the nervous system. While the spleen may influence neuropathic pain, its role has been poorly understood. This study demonstrates that the spleen plays a crucial role in regulating neuropathic pain through the bed nucleus of the stria terminalis (BNST) - paraventricular nucleus of the hypothalamus (PVN) neural circuit in a chronic constriction injury (CCI) mouse model. Splenectomy, splenic denervation, or splenic sympathectomy significantly increased the mechanical withdrawal threshold (MWT) and reduced macrophage infiltration in the dorsal root ganglia (DRG) of CCI mice. Pseudorabies virus injections into the spleen revealed connections to the BNST and PVN in the brain. Chemogenetic inhibition of the BNST-PVN circuit increased macrophage infiltration in the DRG and decreased the MWT; these effects were reversed by splenectomy, splenic denervation, or sympathectomy. These findings underscore the critical role of the spleen, regulated by the BNST-PVN circuit, in neuropathic pain.

**Supplementary Information:**

The online version contains supplementary material available at 10.1007/s12264-025-01454-9.

## Introduction

Neuropathic pain is a significant and widespread health issue, affecting ~7%–10% of the general population [1]. It results from a lesion or disease affecting the somatosensory system and can arise from various conditions such as diabetes, shingles, spinal cord injury, and multiple sclerosis [2]. The chronic nature and complex mechanisms of neuropathic pain contribute to its notable incidence. This type of pain can significantly impair quality of life, leading to issues like sleep disturbances, anxiety, depression, and decreased overall functioning [3, 4]. Managing and treating neuropathic pain presents ongoing challenges in the medical community due to its inadequate response to conventional pain therapies. Moreover, the economic burden is considerable, encompassing both direct healthcare costs and indirect costs such as lost productivity and disability [5]. Consequently, there is an unmet medical need for more effective and targeted therapies to improve patient outcomes and reduce the societal burden of neuropathic pain [6, 7].

The spleen is a critical organ in the immune system, serving several key functions such as filtration of blood (e.g., red blood cell recycling, pathogen removal), immune surveillance (e.g., white pulp, antigen presentation), storage of immune cells (e.g., macrophages, monocytes, lymphocytes), and the production of immune factors (e.g., cytokines and other signaling molecules) [8–12]. Recent research has highlighted the spleen’s role in the modulation of neuropathic pain, demonstrating how the brain encodes pain-state-specific immune responses in the spleen [13]. In addition, interest in the brain–spleen axis in psychiatric and neurological disorders is increasing [14–22]. However, research on the involvement of the brain–spleen axis in neuropathic pain remains limited.

The purpose of this study was to investigate the role of the spleen in neuropathic pain in the mouse model of chronic constriction injury (CCI). First, we examined whether immuno-inflammatory cells are altered in the spleen from CCI mice with neuropathic pain. Second, we investigated whether splenectomy affects neuropathic pain in CCI mice. Third, we explored whether splenic denervation (SD) influences neuropathic pain in CCI mice. Fourth, we assessed whether splenic sympathectomy (SS) by 6-hydroxydopamine (6-OHDA) impacts neuropathic pain in CCI mice. Fifth, we determined which brain regions are affected after the injection of pseudorabies virus (PRV) into the spleen. Finally, we utilized the DREADD (designer receptors exclusively activated by designer drugs) system to study the role of the BNST (bed nucleus of the stria terminalis)-PVN (paraventricular nucleus of the hypothalamus) circuit in neuropathic pain in CCI mice.

## Materials and Methods

### Animals and Surgery

#### Animals

Male C57BL/6J mice, aged 8 to 12 weeks, were procured from Guangdong Zhiyuan Biopharmaceutical Technology Co., Ltd. (Guangzhou, China). The mice were housed in groups of 4-5 per cage. To simulate natural habitat conditions, the environment was maintained at a controlled temperature of 24 ± 2 °C with a 12:12 h light/dark cycle (lights ON at 07:00). The mice had unrestricted access to food and water. Each cage had a constant position throughout the experiment. Animals were randomly assigned to different experimental groups. Sample sizes were estimated based on our previous experience with similar studies [21]. Behavioral experiments and adeno-associated virus (AAV) injection procedures were conducted exclusively on adult male mice. All experimental protocols received approval from two committees: the Institutional Animal Care and Use Committee of the Renmin Hospital of Wuhan University (WDRM-20240201C) and the Laboratory Animal Care and Ethics Committee of Guangdong Laidi Biomedical Research Institute Co., Ltd (2023006–2). In addition, all research complied with the National Institutes of Health Guide for the Care and Use of Laboratory Animals, ensuring the welfare of the animals used in the study. The schematic illustration was drawn using FigDraw tools (www.figdraw.com).

#### Chronic Constriction Injury of Nerves (CCI)

To simulate chronic neuropathic pain resulting from peripheral nerve injury, we applied a CCI model using the unilateral sciatic nerve of mice [23]. The procedure began with anesthetizing each mouse using 1.25% 2,2,2-tribromoethanol (400 mg/kg, T48402, Sigma-Aldrich, St. Louis, USA) administered *via* intraperitoneal injection. Once anesthesia was achieved, the lateral aspect of the left hindlimb was carefully shaved. Each mouse was then positioned ventrally, with its limbs securely taped to prevent involuntary movements during the surgery. The exposed skin area was disinfected with a 0.5% iodine solution before making a precise 1 cm incision. A blunt dissection technique was used to separate the underlying muscles and expose the sciatic nerve. A PVC tube with an inner diameter of ~0.7 mm was placed around the exposed nerve. After this, the muscle layer and skin were meticulously sutured, and the incision site was disinfected again with a 0.5% iodine solution. To minimize the risk of post-operative infection, topical antibiotics were applied to the wound. Following the procedure, each mouse was allowed to recuperate under a warming blanket before being returned to its cage. During the sham surgery, the muscle was opened similarly, but immediately closed after identifying the sciatic nerve.

#### Splenectomy (SE)

The splenectomy and sham surgery were performed under anesthesia using 1.25% 2,2,2-tribromoethanol (400 mg/kg) delivered *via* intraperitoneal injection. In the SE group [24], each mouse was carefully positioned in a right lateral recumbent position. A precise 1-cm incision was made in the abdominal wall under the left costal margin. After dissecting the skin, subcutaneous tissue, muscular layers, and fascia, the spleen was adequately exposed. The peripheral ligament of the spleen was then meticulously separated. Blood vessels and nerves associated with the spleen were securely ligated using 5–0 silk sutures. The spleen was removed by transecting the blood vessels distal to the ligature points. To conclude the procedure, the abdominal muscles and the skin incision were closed using 4–0 silk sutures. During the sham surgery, the abdominal wall was opened similarly, but immediately closed after identifying the spleen. Postoperative pain management did not involve the use of opioids or non-steroidal anti-inflammatory drugs.

#### Splenic Denervation (SD)

The mice were anesthetized using 1.25% 2,2,2-tribromoethanol (400 mg/kg). Access to the peritoneal cavity was established through a midline abdominal incision [18]. The spleen was isolated from the peritoneal cavity using forceps, ensuring clear exposure of its three main vascular supplies. To protect the peritoneal cavity and adjacent organs, a moistened cotton pad was strategically placed during the procedure. Under a dissecting microscope, absolute ethanol was meticulously applied to the exposed vascular trees using cotton tips. This application was repeated seven times, each for 5–10 s at intervals of 5 s. The goal was to deplete the splenic nerve fibers running along these vascular trees. Precise care was taken to prevent excessive dripping of ethanol, which could potentially cause visible vessel spasms, leading to irreversible damage to the blood vessels and resulting in splenic necrosis and complete organ absorption. In the sham-operated mice, the surgical procedures were identical, except that saline (pH 7.4) was used in place of absolute ethanol. Following the denervation surgery, the animals were allowed a recovery period of 2 weeks before proceeding with further study processes.

#### Splenic Sympathectomy (SS)

Building upon the foundational knowledge of the SS technique from prior research [18, 25], we aimed to perform splenic sympathetic denervation while preserving the sympathetic innervation in other anatomical regions as much as possible. The SS denervation surgery followed established protocols. Mice were anesthetized using an intraperitoneal injection of 1.25% 2,2,2-tribromoethanol (400 mg/kg). Once anesthetized, a midline abdominal incision was made to access the abdominal cavity. The spleen was then bluntly separated from the cavity to expose its three major vascular networks. Cotton balls saturated with a solution of 6-hydroxydopamine (6-OHDA, 2 mg/mL, prepared using a 0.2% ascorbic acid saline solution; Shanghai Yuanye Bio-Technology Co., Ltd, #S30042, Shanghai, China) were applied to the exposed spleen. The soaking process, which lasted for 10 s each time, was repeated seven times to ablate the sympathetic nerve fibers running along these vascular networks. To prevent leakage of the 6-OHDA solution into the abdominal cavity, wet cotton pads were used to isolate the spleen from other organs during the soaking process. In the vehicle-operated (Sham) mice, an identical surgical procedure was performed, but with the application of only the 0.2% ascorbic acid saline solution. Following the surgery, all mice were allowed a recovery period of two weeks before proceeding with further study processes.

### Pseudorabies Virus Injection into the Spleen

The Pseudorabies virus (PRV-CAG-EGFP, BC-PRV-531, Brain Case, Shenzhen, China, ≥5.0 × 10^9^ PFU/mL) was prepared for injection into adult mice [26], which were anesthetized using 1.25% 2,2,2-tribromoethanol (400 mg/kg). The spleen was accessed *via* a midline incision into the peritoneal cavity. Using pulled glass micropipettes (3.5", 3000203G/X, Drummond Scientific, Philadelphia, USA), the prepared virus was injected into the upper, middle, and lower extremities of the spleen (100 nL/site) at a controlled rate of 1 nL/s using a microinjection system (R480, RWD Life Science Co., Ltd, Shenzhen, China). Upon completion of the injections, the surgical wound was meticulously closed using standard sutures. Five days post-surgery, the designated mice were euthanized, and their brains were extracted for further examination. The collected brains were processed to identify PRV-EGFP^+^ neurons in various regions. The procedure involved initially fixing the brains overnight in 4% paraformaldehyde (PFA), followed by dehydration in 30% sucrose at 4°C for 2–3 days. Subsequently, the brains were coronally cut at 50 μm on a freezing microtome (Leica 1950, Wetzlar, Germany). Fluorescence images of these sections were captured under a microscope (Keyence, Osaka Prefecture, Japan) to facilitate the investigation of PRV-EGFP^+^ neurons.

### Stereotaxic Injection Procedure

C57BL/6J mice were anesthetized *via* intraperitoneal (i.p.) administration of 1.25% 2,2,2-tribromoethanol (400 mg/kg) and secured in a stereotaxic apparatus (RWD Life Science Co., Ltd, Shenzhen, China). To prevent ocular desiccation during the procedure, ophthalmic ointment was applied. The animals were placed on a heating pad to maintain their body temperature within the range of 35°C to 37°C. Before cranial surgery, scalp hair was removed, and the skin was disinfected with iodine and medical alcohol. A careful incision was made in the scalp to reveal the underlying skull, and adhering connective tissue was gently cleared using cotton swabs. Under the guidance of a surgical microscope, craniotomy holes, ~1 mm in diameter, were drilled into the skull. Stereotaxic coordinates were defined as dorsal-ventral (DV) from the brain surface, anterior-posterior (AP) from bregma, and medio-lateral (ML) from the midline (in mm). Injection targets were identified at specific brain regions with their respective coordinates (AP, ML, DV in mm): BNST (AP −0.1, ML ± 0.8, DV −4.0) and PVN (AP −0.8, ML ± 0.2, DV −4.8). To prevent virus spillage from the injection site, the micropump was left in place for 5 min post-injection. Following this, the incision was sutured and sterilized. After surgery, each animal was placed on a heating pad until it regained consciousness and exhibited normal activities, after which it was returned to its cage.

### Viral Injections and Chemogenetic Manipulation

Utilizing pulled glass micropipettes (3.5", 3000203G/X, Drummond Scientific, Philadelphia, USA), the virus was intracranially injected (200 nL) at a controlled rate of 1 nL/s using a microinjection system (R480, RWD Life Science Co., Ltd, Shenzhen, China) [27]. To prevent virus spillage from the injection site, the micropump was left in place for an additional 5 min following the injection. Subsequently, the incision was sutured and sterilized. Post-operative care involved placing the animals on heating pads until they regained consciousness and resumed normal activities, after which they were returned to their cages. For the chemogenetic activation or inhibition of the BNST-PVN pathway in C57BL/6J mice, bilateral injections into the BNST were conducted using either AAV2/9-hSyn-DIO-hM4D(Gi)-mCherry-WPRE-pA (S0193-9, Shanghai Taitool Bioscience Co., Ltd, titer: 1 × 10^13^ VG/mL, Shanghai, China), AAV2/9-hSyn-DIO-hM3D(Gq)-mCherry-WPRE-pA (S0918-9, Shanghai Taitool Bioscience Co., Ltd, titer: 1 × 10^13^ VG/mL, Shanghai, China), or AAV2/9-hSyn-DIO-mCherry-WPRE-pA (S1138-9, Shanghai Taitool Bioscience Co., Ltd, titer: 1 × 10^13^ VG/mL, Shanghai, China). Meanwhile, the PVN was injected with AAV2/2Retro-hSyn-Cre-WPRE-pA (S0278-2R, Shanghai Taitool Bioscience Co., Ltd, titer: 1 × 10^13^ VG/mL, Shanghai, China).

For combinatorial experiments, surgical procedures such as SE, SD, or SS were performed two days prior to viral injections. Subsequently, AAV2/9-hSyn-DIO-hM4D(Gi)-mCherry-WPRE-pA was injected into the BNST, and a mixture of retro-Cre and CTB488 was injected into the PVN. After a 14-day recovery period, CCI surgery is performed.

### Chemogenetic Protocols

Mice were randomly assigned to receive either an intraperitoneal injection of 2 mg/kg clozapine-*N*-oxide (CNO, ab141704, Abcam, Cambridge, USA) or an equivalent volume of saline. To evaluate the effects of chemogenetic activation or inhibition of the BNST-PVN neural circuit, MWTs were assessed 1 h post-injection.

### Mechanical Withdrawal Threshold (MWT)

The MWT was assessed using an electronic Von Frey Aesthesiometer (Bioseb, Vitrolles, France) [28]. Each mouse was placed on an elevated mesh platform within an acrylic cage and given a 30 min acclimation period before testing. An elastic spring-type tip attached to a hand-held force transducer was used to stimulate the plantar surface of the right hind paw. Each mouse underwent the test five times, with a resting interval of 3 min between each test. The MWT was defined as the average force (in grams) that elicited a withdrawal response from five consecutive measurements. This measure is also known as the Paw Withdrawal Threshold. Both terms are interchangeable and describe mechanical allodynia.

### Open Field Test (OFT)

The OFT was used to assess the locomotor activity of the mice. Before the test, the mice were acclimated to the testing environment for at least 60 min. Subsequently, each mouse was placed in the center of a polystyrene enclosure (45 cm × 45 cm × 35 cm). During the test, the mouse was allowed to roam freely within the enclosure for 10 min, and its movements were recorded. The "center area" was defined as a 22.5 cm × 22.5 cm rectangular area located at the midpoint of the field. Following each trial, the open field was cleaned with alcohol-free antiseptic wipes to ensure a neutral environment for every mouse. Video recordings of the animal’s track were analyzed in real-time using ANY-maze software (Stoelting, Illinois, USA). This analysis extracted key data, such as the total distance traversed (measured in meters), which served as an indicator of locomotor activity, and the maximum speed (measured in meters per second) achieved by each mouse.

### Flow Cytometry

Upon administering anesthesia to the mice with 1.25% 2,2,2-tribromoethanol (400 mg/kg, i.p.), their spleens were extracted and homogenized using a 70 μm cell strainer (#251200, Sorfa, Huzhou, China) [29]. Following this, the spleens were rinsed with a flow buffer composed of 50 mL phosphate buffer saline (PBS), 1 mL fetal bovine serum (FSX3000, Sorfa, Huzhou, China), and 200 μL ethylene diamine tetraacetic acid (Thermo Fisher Scientific, 15575020, Waltham, MA, USA). Red blood cells (RBCs) in the sample were lysed using RBC Lysis Buffer (BioLegend, 420302, CA, USA), and the mixture was then centrifuged. The cell pellet was resuspended in the flow buffer to yield a single-cell suspension. This suspension was incubated with fluorescence-labeled antibodies in a 96-well U-bottom plate at 4°C in the dark for 1 h. Subsequently, the Aria FACS Fusion II cytometer (BD Bioscience, New York, USA) was utilized for detection, and FACS analyses were conducted using FlowJo software. The antibodies for flow cytometry were: Zombie Aqua™ Fixable Viability Kit (Biolegend, 423101, CA, USA), FITC anti-mouse F4/80 (Biolegend, 123107, CA, USA), anti-CD11b-APC (BD Bioscience, 553312, NY, USA), and anti-mouse CD16/32 (Biolegend, 101320, CA, USA).

### Tissue Collection

Mice were anesthetized with 1.25% 2,2,2-tribromoethanol (400 mg/kg, i.p.) and subsequently underwent transcardial perfusion with PBS, which was followed by perfusion with 4% PFA. Post-perfusion, brain and left dorsal root ganglia (DRG) samples from the L4–6 segments were carefully dissected. Following dissection, brain samples were dehydrated in a 30% sucrose solution at 4°C until they were fully submerged. These dehydrated brains were then coronally cut at 50 μm on a freezing microtome (Leica 1950, Wetzlar, Germany). Fluorescence images of the sections were obtained using a microscope (Keyence, Osaka Prefecture, Japan), providing critical visual data for further analysis.

### Immunofluorescence (IF)

The DRG was fixed in 4% paraformaldehyde for 2 h, followed by 2 h of dehydration in 20% sucrose solution. The tissue was cut at 10 μm on a freezing microtome (Leica 1950, Wetzlar, Germany). These sections were then treated with 3% Triton X-100 at room temperature for 15 min, rinsed thoroughly with PBS, and blocked with a rapid blocking solution (Beyotime, #P0260, Shanghai, China) for an additional 15 min. Primary antibodies [Iba-1 (Cell Signaling Technology, #17198S, 1:200, Boston, USA), Iba-1 (Synaptic Systems, #Gp311H9, 1:500, Goettingen, Germany), NeuN (Sigma-Aldrich, #ABN90P, 1:200, USA), calcitonin gene-related peptide (CGRP, Cell Signaling Technology, #14959S, 1:200, Boston, USA), cFos (ServiceBio, #GB12069, 1:500, Wuhan, China), CD86 (Cell Signaling Technology, #19589S, 1:200, Boston, USA), and Ki67 (ServiceBio, #GB121141, 1:500, Wuhan, China) were diluted in the blocking solution and incubated overnight at 4°C. Post-primary antibody incubation, sections underwent three successive 5-min washes with PBS, after which they were incubated with fluorescent secondary antibodies [goat anti-guinea pig IgG H&L (Alexa Fluor^®^ 594) (#ab150188, Abcam, Cambridge, USA), goat anti-rabbit IgG H&L (Alexa Fluor® 594) (#ab150080, Abcam, Cambridge, USA), goat anti-rabbit IgG H&L (Alexa Fluor® 488) (#ab150077, Abcam, Cambridge, USA), and goat anti-mouse IgG H&L (Alexa Fluor® 488) (#ab150113, Abcam, Cambridge, USA)]. These secondary antibodies were diluted in PBS and applied to the sections at room temperature for 2 h. Following another set of three 5-min washes with PBS, the sections were treated with DAPI (Beyotime, #AR1176, Shanghai, China) for 5 min. After rinsing the sections thrice with PBS, they were mounted for observation. A fluorescence microscope was used to capture images and detect the presence of NeuN^+^, CGRP^+^, and Iba-1^+^ cells in the DRG. Cell counting was subsequently performed utilizing ImageJ software for analysis.

### Statistical Analysis

Data are presented in the format of mean ± SEM. Before proceeding with further analysis, normality was assessed for each dataset using the Shapiro–Wilk test. For datasets that conformed to a normal distribution, parametric tests such as unpaired *t*-tests, one-way or two-way ANOVA were applied to analyze the variance and group differences. The repeated measures ANOVA was specifically used for analyzing the MWT, provided the data followed a normal distribution. Sphericity was assessed using Mauchly's test, and a Greenhouse-Geisser correction was applied when sphericity was violated. All statistical analyses were conducted using GraphPad Prism 9 (GraphPad Software Inc., San Diego, CA, USA).

## Results

### CCI Leads to Increased Macrophages in the Spleen and DRG

**Fig. 1:**
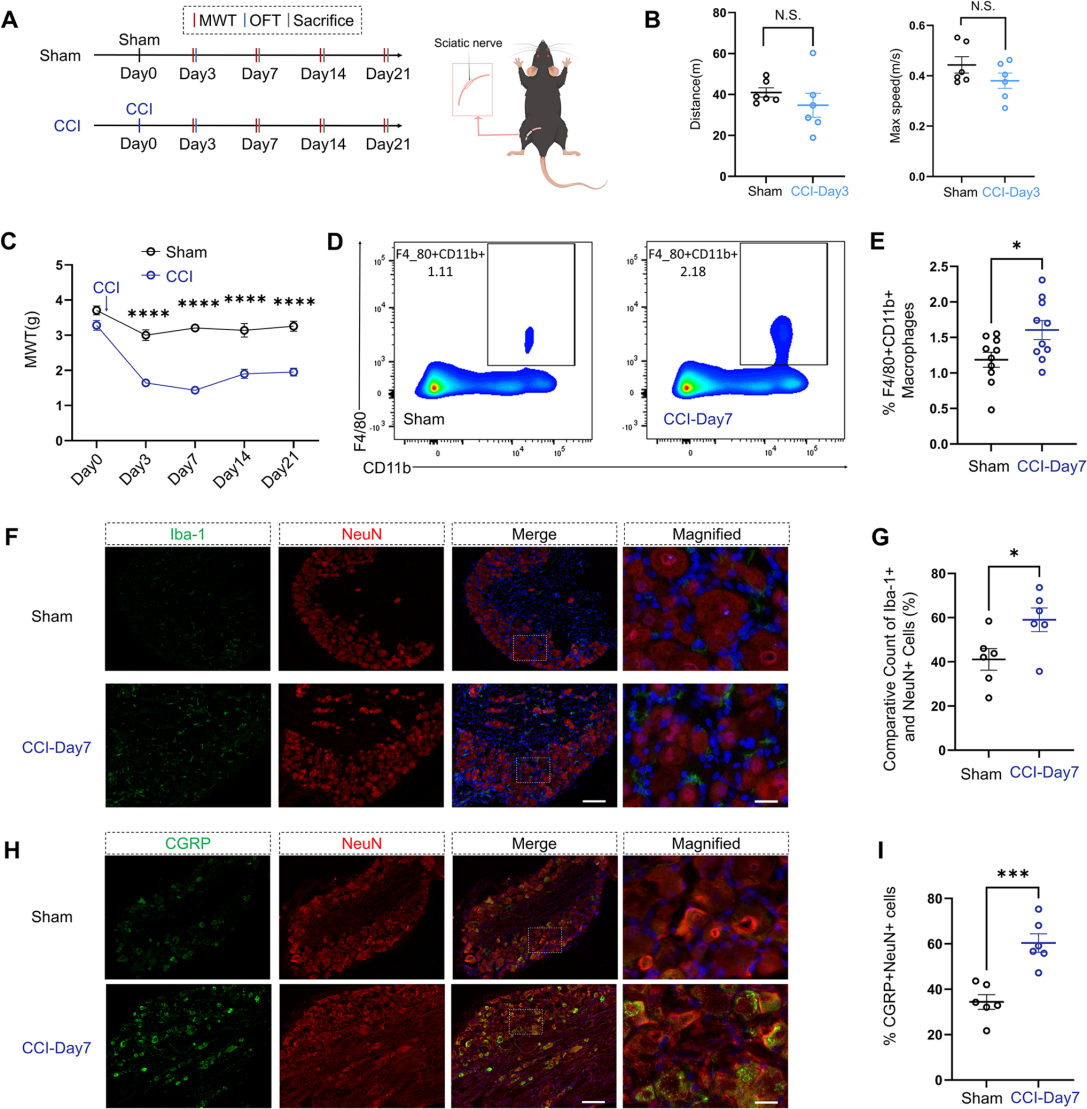
Characterization of the CCI mouse model. **A** Schematic representation of the CCI procedure performed on the sciatic nerve of mice. **B** Experimental timeline for behavioral testing in sham and CCI groups. MWT: mechanical withdrawal threshold, OFT: open field test. Quantification of the total distance traveled in the OFT shows no significant difference between sham and CCI mice on day 3 post-CCI (two-tailed unpaired Student’s *t*-test, *t* = 0. 9985, *df* = 10, *P =* 0.3462; *n* = 6 per group). N.S.: no significant difference. Quantification of the maximum speed in the OFT shows no significant difference between sham and CCI mice on day 3 post-CCI (two-tailed unpaired Student’s *t*-test, *t* = 1.409, *df* = 10, *P =* 0.1891; *n* = 6 per group). N.S.: no significant difference. **C** MWT measurements over time in sham and CCI mice. CCI mice show a significant decrease in MWT on days 3, 7, 14, and 21 post-CCI compared to Sham mice (repeated measures two-way ANOVA, time: *F*_(4, 80)_ = 31.22, *P <* 0.0001; CCI: *F*_(1, 20)_ = 162.8, *P <* 0.0001; interaction: *F*_(4, 80)_ = 7.969, *P <* 0.001, *n* = 11 per group, ****P < 0.0001). **D, E** Flow cytometry analysis of spleen macrophages (F4/80^+^CD11b^+^ cells) showing an increased proportion of macrophages in CCI mice compared to Sham mice (two-tailed unpaired Student’s *t*-test, *t* = 2.456, *df* = 18, *P =* 0.0244, *n* = 10 per group). *P < 0.05. **F, G** Immunofluorescence analysis of dorsal root ganglia (DRG) demonstrating a ratio of Iba-1^+^ macrophages to NeuN^+^ neurons in CCI mice compared to sham mice (two-tailed unpaired Student’s *t*-test, *t* = 2.482, *df* = 10, *P =* 0.0324, *n* = 6 per group, *P < 0.05). Scale bars, 100 µm, magnified 20 µm. **H, I** Immunofluorescence analysis showing increased CGRP^+^ neuron counts in the DRG of CCI mice compared to Sham mice (two-tailed unpaired Student’s *t*-test, *t* = 5.014, df = 10, *P =* 0.0005, *n* = 6 per group, ***P < 0.001.). Scale bars, 100 µm, magnified 20 µm. Data are presented as the mean ± SEM.

Using established methods [23], we successfully created a CCI mouse model without affecting the motor function of the mice (Figs 1A, B, and S1A). Compared to the sham control group, the CCI group exhibited a significant decrease in the MWT, a hallmark of mechanical allodynia, on postoperative days 3, 7, 14, and 21 (Fig. 1C). Further statistical analysis revealed significant differences over time, between groups, and in their interaction. Flow cytometry analysis of the spleen showed an elevated proportion of macrophages (F4/80^+^ and CD11b^+^) in the CCI-day 7 group compared to the sham group (Fig. 1D, E). The difference was statistically significant. Immunofluorescence detection in the left DRG from lumbar segments 4–6 revealed a significant increase in the ratio of macrophages (Iba-1^+^) to neuronal cells (NeuN^+^) in the CCI group (Fig. 1F, G). Furthermore, the ratio of M1 phenotype (CD86^+^) to macrophages (Iba-1^+^) in the DRG was also elevated (Fig. S2A, B). However, macrophage proliferation (Ki67^+^) and neuronal activity (cFos^+^) in the DRG showed no significant differences (Fig. S2C–F). In addition, there was a notable increase in the proportion of pain-related protein (CGRP)-positive neurons in the DRG of the CCI-day 7 group (Fig. 1H, I). This trend continued on day 14 and day 21 (Fig. S1B, C). Interestingly, the proportion of CGRP^+^ neurons (CGRP^+^NeuN^+^ cells/NeuN^+^ cells) in the DRG was positively correlated with the proportion of macrophages (Iba1^+^ cells/NeuN^+^ cells) in the DRG across both groups (Fig. S1D). A similar correlation trend was found between macrophages (F4/80^+^CD11b^+^ cells) in the spleen and macrophages (Iba1^+^ cells/NeuN^+^ cells) in the DRG (Fig. S1E).

Based on these observations of the most substantial reduction in MWT and the highest CGRP^+^/NeuN^+^ ratio in the DRG occurring in the CCI group on day 7, we selected the seventh day after the CCI procedure as our research study endpoint.

### Splenectomy Mitigates Macrophage Infiltration within the DRG of CCI Mice

**Fig. 2:**
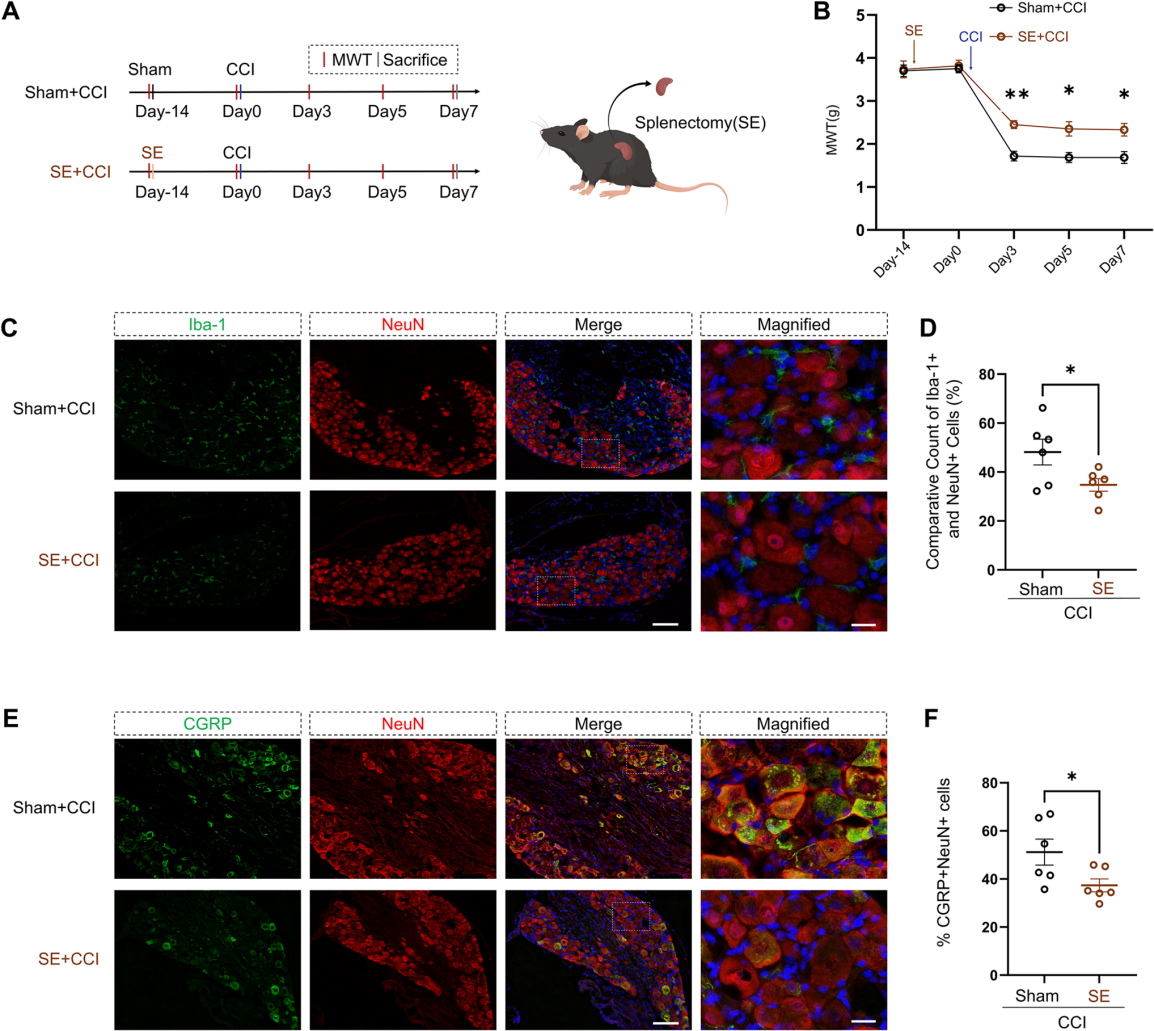
Effect of splenectomy (SE) on CCI mice. **A** Experimental timeline for CCI and SE procedures in mice. MWT: Mechanical Withdrawal Threshold. Schematic representation of SE performed on mice. **B** MWT measurements over time in Sham + CCI and SE + CCI mice. SE mice show significantly higher MWT on days 3, 5, and 7 post-CCI compared to Sham + CCI mice (repeated measures two-way ANOVA, time: *F*_(2.438, 24.38)_ = 95.40, *P <* 0.0001; SE: *F*_(1, 10)_ = 22.29, *P =* 0.0008; interaction: *F*_(4, 40)_ = 3.282, *P =* 0.0204; *n* = 6 per group). *P < 0.05, **P < 0.01. **C, D** Immunofluorescence analysis of the DRG showing a lower ratio of Iba-1^+^ macrophages to NeuN^+^ neurons in SE + CCI mice compared to Sham + CCI mice (two-tailed unpaired Student’s *t*-test, *t* = 2.286, *df* = 10, *P =* 0.0453, *n* = 6 per group). *P < 0.05. Scale bars, 100 µm, magnified 20 µm. **E, F** Immunofluorescence analysis of the DRG showing decreased CGRP^+^ neuron counts in SE + CCI mice compared to Sham + CCI mice (two-tailed unpaired Student’s *t*-test,* t* = 2.292, df = 10, *P =* 0.0449; *n* = 6 per group). *P < 0.05. Scale bars, 100 µm, magnified 20 µm. Data are presented as the mean ± SEM.

To elucidate the role of the spleen in CCI, we performed the SE procedure [24] 14 days before inducing CCI. This was done to assess the influence of the spleen on neuropathic pain resulting from CCI (Fig. 2A). The SE + CCI group showed a significant increase in MWT on postoperative Day 3 (*P =* 0.0032), Day 5 (*P =* 0.0475), and Day 7 (*P =* 0.0471) compared to the Sham + CCI group, indicating a statistically significant difference (Fig. 2B).

Immunofluorescence analysis revealed that the ratio of macrophages to neuronal cells in the DRG was significantly lower in the SE + CCI group compared to the Sham + CCI group (Fig. 2C, D). In addition, there was a marked decrease in the proportion of CGRP-positive neurons in the SE + CCI group (Fig. 2E, F).

### Splenic Denervation Attenuates Macrophage Infiltration in the DRG and Reduces Macrophages within the Spleen

**Fig. 3:**
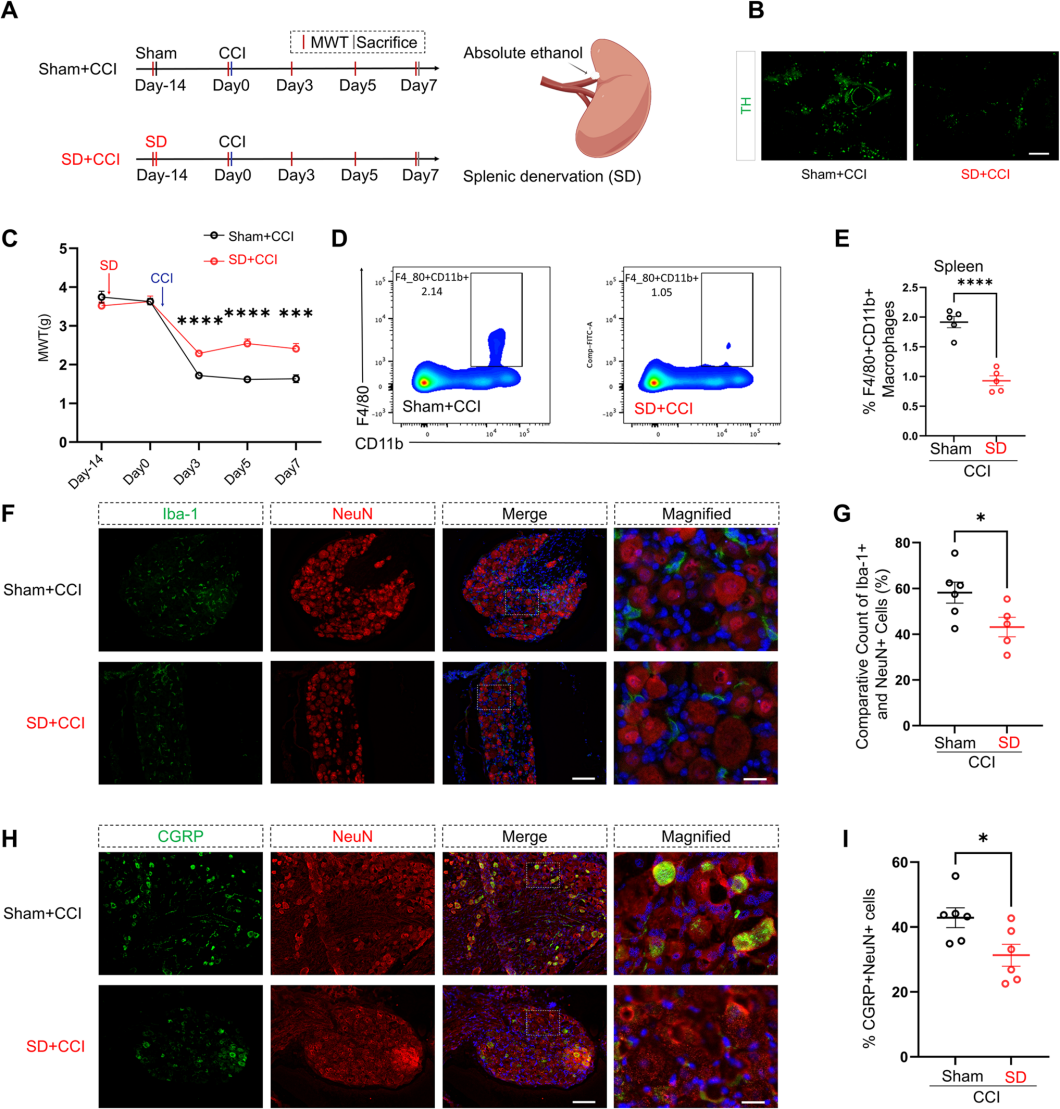
SD alleviates neuropathic pain in CCI mice. **A** Experimental timeline for CCI and SD procedures. MWT: mechanical withdrawal threshold. Schematic representation of SD performed using absolute ethanol. **B** Immunofluorescence analysis of the spleen showing a reduction in TH^+^ area in SD + CCI mice compared to Sham + CCI mice. Scale bar, 100 µm. **C** MWT measurements over time in Sham + CCI and SD + CCI mice. SD mice show significantly higher MWT on days 3, 5, and 7 post-CCI than Sham + CCI mice (repeated measures two-way ANOVA, time: *F*_(3.180, 63.60)_ = 142.1, *P <* 0.0001; SD: *F*_(1, 20)_ = 28.00, *P <* 0.0001; interaction: *F*_(4, 80)_ = 11.65, *P <* 0.0001; *n* = 11 per group). **P < 0.01. **D, E** Flow cytometry analysis of spleen macrophages (F4/80^+^CD11b^+^ cells) showing a reduced proportion of macrophages in SD + CCI mice compared to Sham + CCI mice (two-tailed unpaired Student’s *t*-test, *t* = 7.858, *df* = 8, *P <* 0.0001, *n* = 5 per group). ****P < 0.0001. **F, G** Immunofluorescence analysis of the DRG showing a lower ratio of Iba-1^+^ macrophages relative to NeuN^+^ neurons in SD + CCI mice compared to Sham + CCI mice (two-tailed unpaired Student’s *t*-test, *t* = 2.362, *df* = 9, *P =* 0.0425; sham + CCI, *n* = 6; SD + CCI, *n* = 5). *P < 0.05. Scale bars, 100 µm, magnified 20 µm. **H, I** Immunofluorescence analysis of the DRG showing decreased CGRP^+^ neuron counts in SD + CCI mice compared to Sham + CCI mice (two-tailed unpaired Student’s *t*-test, *t* = 2.539, *df* = 10, *P =* 0.0294; *n* = 6 per group). *P < 0.05. Scale bars, 100 µm, magnified 20 µm. Data are presented as the mean ± SEM.

Given the direct innervation of the spleen by the splenic nerve, we investigated its role in CCI by performing the SD procedure using anhydrous ethanol [18]. This approach aimed to further elucidate the impact of the splenic nerve on mechanical hypersensitization in CCI mice (Fig. 3A). Immunofluorescence results confirmed successful splenic denervation, showing a significant decrease in the sympathetic nerve marker tyrosine hydrolase (TH)-positive area within the spleens of the SD + CCI group (Fig. 3B).

Compared to the Sham + CCI group, the SD + CCI group exhibited a significant increase in MWT on postoperative day 3 (*P <* 0.0001), day 5 (*P <* 0.0001), and day 7 (*P =* 0.0010), showing a statistically significant difference (Fig. 3C). Flow cytometry analysis showed a significant reduction in the proportion of macrophages (F4/80^+^ and CD11b^+^ cells) within the spleens of the SD + CCI group mice compared to the Sham + CCI group (Fig. 3D, E).

Immunofluorescence results further showed diminished macrophage infiltration within the DRG of the SD + CCI group (Iba-1^+^) (Fig. 3F, G), accompanied by a significant decline in the proportion of CGRP-positive neurons (Fig. 3H, I). These findings suggest that SD could potentially alleviate the reduction in MWT induced by CCI.

### Splenic Sympathectomy Negates Macrophage Accumulation in the Spleen and DRG Macrophage Infiltration

**Fig. 4:**
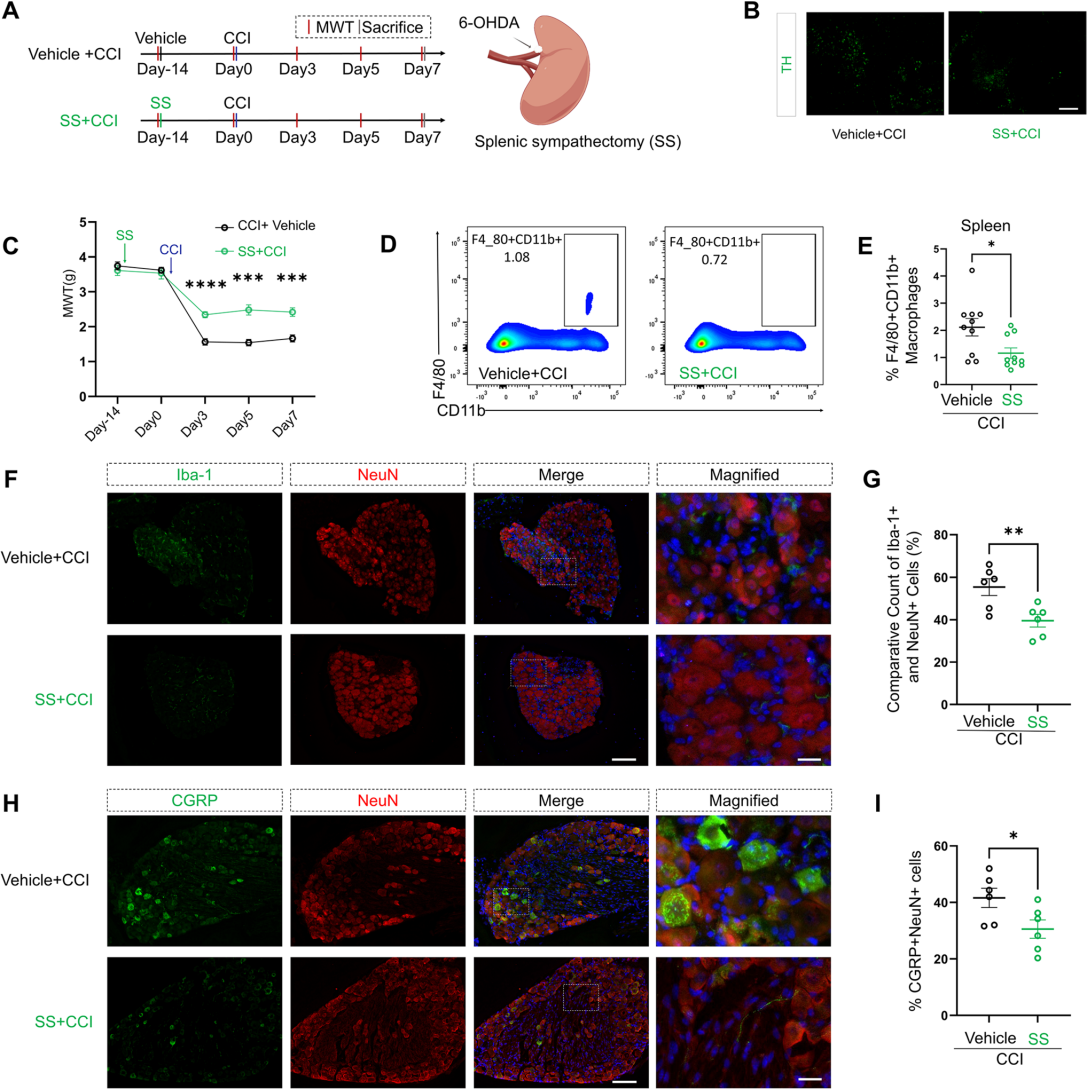
SS mitigates CCI-induced neuropathic pain and modulates immune cell populations. **A** Experimental timeline. Mice underwent either SS or sham surgery, followed by a 14-day recovery period. Subsequently, CCI surgery was performed. The Mechanical Withdrawal Threshold (MWT) was measured on days 3, 5, and 7 post-CCI surgery. Schematic representation of the SS procedure. **B** Immunofluorescence images of the spleen showing a significant decrease in TH^+^ area in SS mice, verifying the success of the SS. Scale bar, 100 µm. **C** The MWT shows a significant increase in SS mice on days 3 and 7 post-CCI surgery compared to sham-operated mice (repeated measures two-way ANOVA, time: *F*_(2.888, 57.77)_ = 122.2, *P <* 0.0001; SS: *F*_(1, 20)_ = 28.77, *P <* 0.0001; interaction: *F*_(4, 80)_ = 10.26, *P <* 0.0001; *n* = 11 per group). **P < 0.01. **D, E** Representative flow cytometry plots showing the population of F4/80^+^ and CD11b^+^ cells in the spleen for each group. Quantification of F4/80^+^ and CD11b^+^ macrophages in the spleen shows a significant reduction in the SS group compared to the sham group (two-tailed unpaired Student’s *t*-test, *t* = 2.544, *df* = 18, *P =* 0.0204, *n* = 10 per group). *P < 0.05. **F, G** Immunofluorescence images of the DRG showing a lower ratio of Iba-1^+^ cells to NeuN^+^ cells in SS mice compared to sham-operated mice. Quantification of Iba-1^+^ cells relative to NeuN^+^ cells in the DRG shows a significant reduction in SS mice (two-tailed unpaired Student’s *t*-test, *t* = 3.192, *df* = 10, *P =* 0.0096; *n* = 6 per group). **P < 0.01. Scale bars, 100 µm, magnified 20 µm. **H, I** Immunofluorescence images of the DRG showing a decreased ratio of CGRP^+^ cells to NeuN^+^ cells in SS mice compared to sham-operated mice. Quantification of CGRP^+^ cells relative to NeuN^+^ cells in the DRG shows a significant reduction in SS mice (two-tailed unpaired Student’s *t*-test, *t* = 2.351, *df* = 10, *P =* 0.0406; *n* = 6 per group). *P < 0.05. Scale bars, 100 µm, magnified 20 µm. Data are presented as the mean ± SEM.

To determine whether the sympathetic nerves within the splenic nerve play a pivotal role, we conducted the SS procedure using 6-OHDA [18, 25] and performed CCI surgery 14 days post-denervation (Fig. 4A). Immunofluorescence results showed a considerable reduction in the area positive for the sympathetic nerve marker TH, indicating successful denervation in the spleens of the SS + CCI group (Fig. 4B).

The MWT significantly increased in the SS + CCI group compared to the vehicle + CCI group on postoperative day 3 (*P <* 0.0001), day 5 (*P =* 0.0002), and day 7 (*P =* 0.0006), statistically significant differences (Fig. 4C). Flow cytometry analysis demonstrated that the proportion of macrophages (F4/80^+^ and CD11b^+^ cells) in the spleens of SS + CCI group mice were significantly decreased compared to the vehicle + CCI group (Fig. 4D, E). Furthermore, there was a notable reduction in macrophage infiltration in the DRG of the SS + CCI group (Fig. 4F, G), and the ratio of M1 phenotype (CD86^+^) to macrophages (Iba-1^+^) in the DRG was also reduced (Fig. S3A, B). A significant decrease was also recorded in the proportion of CGRP-positive neurons in the DRG of the SS + CCI group (Fig. 4H, I).

### Spleen Injection of PRV Retrogradely Identifies Connections to the BNST and PVN

To unravel the neural connections between specific brain nuclei and the spleen, we utilized a reliable retrograde neural tracer, PRV-EGFP [30]. By injecting PRV-EGFP into the spleen, we aimed to identify the associated neural nuclei.

Following the administration of PRV into the spleen and allowing five days for tracing (Fig. S4A), we captured images of whole-brain sections of the mouse brain. These images depicted the presence of EGFP-labeled neurons in several regions, including the BNST (Bregma: − 0.22 mm), the Central Amygdala (CeA, Bregma: − 1.06 mm), PVN (Bregma: − 1.06 mm), Lateral Hypothalamus (LH, Bregma: − 2.06 mm), Lateral parabrachial nucleus (LPB, Bregma: − 5.02 mm), the alpha parts of gigantocellular reticular nucleus (GiA, Bregma: − 6.12 mm), magnocellular part of the medial vestibular nucleus (MVeMC, Bregma: − 6.64 mm) and Adrenergic cell group C3 (C3, Bregma: − 6.72mm) (Fig. S4B).

Despite examining other organs such as the heart, lungs, liver, kidneys, and colon, no EGFP-labeled neurons were observed (Fig. S4C). This finding suggests that the splenic nerve forms a direct connection with the brain, rather than linking *via* other visceral nerves.

### Inhibiting the BNST-PVN Neural Circuit Bolsters Macrophage Infiltration within the DRG, Whereas Activation Induces an Antithetical Outcome

Previous studies have highlighted the critical role of the BNST and PVN in visceral pain provoked by maternal separation [31]. However, their function in somatic pain has yet to be explored. Therefore, our investigation sought to ascertain if the BNST-PVN neural circuit also plays a fundamental regulatory role in somatic pain.

**Fig. 5:**
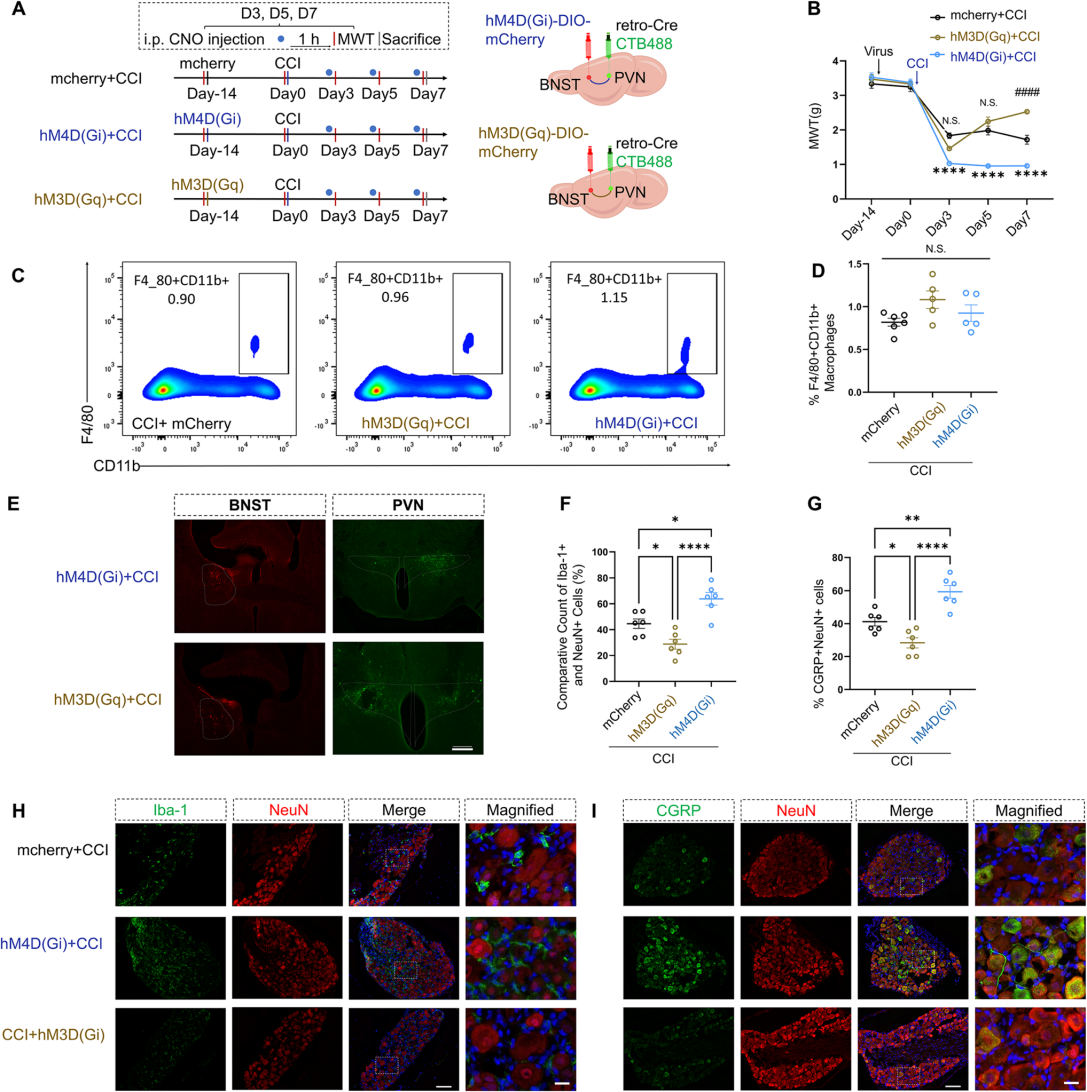
The BNST-PVN neural circuit's role in neuropathic pain induced by CCI. **A** Experimental timeline. Mice receive stereotactic injections into the BNST with AAV2/9-hSyn-DIO-hM3D(Gq)-mCherry-WPRE-pA, AAV2/9-hSyn-DIO-hM4D(Gi)-mCherry-WPRE-pA, or AAV2/9-hSyn-DIO-mCherry-WPRE-pA viruses. In addition, the PVN region was injected with a mixture of retro-Cre and CTB488. After a 14-day recovery period, all three groups underwent CCI surgery. Schematic representation of the viral injection sites in the BNST and PVN. **B** The MWT shows a significant increase in hM3D(Gq) mice on day 7 post-CCI surgery, whereas hM4D(Gi) mice showed a significant decrease on days 3, 5, and 7 post-CCI surgery compared to mCherry controls (repeated measures two-way ANOVA, time: *F*_(4, 132)_ = 273.9, *P <* 0.0001; virus: *F*_(2, 33)_ = 52.22, *P <* 0.0001; interaction: *F*_(8, 132)_ = 17.64, *P <* 0.0001; *n* = 12 per group). ^####^*P <* 0.0001. ***P < 0.001, ****P < 0.0001. N.S.: no significant difference. **C, D** Representative flow cytometry plots showing the population of F4/80^+^ and CD11b^+^ macrophages in the spleen for each group. Quantification of F4/80^+^ and CD11b^+^ macrophages in the spleen reveal no significant differences among the mCherry, hM3D(Gq), and hM4D(Gi) groups (one-way ANOVA, *F*_(2, 13)_ = 2.695, *P =* 0.1049; mCherry + CCI, *n* = 6, hM3D(Gq) + CCI, *n* = 5, hM4D(Gi) + CCI, *n* = 5, N.S.: no significant difference). **E** Immunofluorescence images showing fluorescently labeled neurons in both the BNST and PVN, confirming accurate viral injection sites and expression. Scale bar, 100 µm. **F, H** Immunofluorescence images of the DRG showing Iba-1^+^ (green) and NeuN^+^ (red) cells. Quantification of Iba-1^+^ cells relative to NeuN^+^ cells in the DRG shows fewer Iba-1^+^ cells in hM3D(Gq) mice and more in hM4D(Gi) mice compared to mCherry controls (one-way ANOVA, *F*_(2, 15)_ = 17.73, *P =* 0.0001; *n* = 6 per group). *P < 0.05, ****P < 0.0001. Scale bars, 100 µm, magnified 20 µm. **G, I** Immunofluorescence images of the DRG showing CGRP^+^ (green) and NeuN^+^ (red) cells. Quantification of CGRP^+^ cells relative to NeuN^+^ cells in the DRG show a similar trend, with fewer CGRP^+^ cells in hM3D(Gq) mice and more in hM4D(Gi) mice compared to mCherry controls (one-way ANOVA, *F*_(2, 15)_ = 23.63, *P <* 0.0001;* n* = 6 per group). *P < 0.05, **P < 0.01, ****P < 0.0001. Scale bars, 100 µm, magnified 20 µm. Data are presented as the mean ± SEM.

By implementing DREADDs, we successfully conducted chemogenetic manipulation. Specialized neural circuits were targeted through the Cre-loxP recombinase system. Following simultaneous viral injection into the BNST and PVN (using DIO-mCherry as a control virus, hM3D(Gq)-DIO-mCherry for activation, hM4D(Gi)-DIO-mCherry for inhibition, retro-Cre for retrograde infection across single synapses, and cholera toxin subunit B conjugated with Alexa Fluor 488 (CTB488) for localization), CCI was performed 14 days post-injection (Fig. 5A). We detected mCherry fluorescent-labeled neurons in the BNST region and CTB488 fluorescent-labeled neurons in the PVN area, thus verifying the precision of our viral injection sites and successful expression (Fig. 5E).

One hour after intraperitoneal injection of CNO, MWTs were measured. Compared to the mCherry + CCI group, the hM3D(Gq) + CCI group (where the neural circuit was activated) exhibited a significant increase in MWT on day 7 post-CCI surgery (*P <* 0.0001) with no considerable differences noted on days 3 (*P =* 0.1385) and 5 (*P =* 0.6635). In the hM4D(Gi) + CCI group, where the neural circuit was inhibited, mechanical allodynia displayed a noteworthy decline on days 3, 5, and 7 post-CCI surgery (*P <* 0.0001 each). The overall variance was statistically significant (Fig. 5B).

Flow cytometry analysis showed no notable differences in the proportion of macrophages (F4/80^+^ and CD11b^+^ cells) in the spleens of hM3D(Gq) + CCI and hM4D(Gi) + CCI mice when compared to the control group (Fig. 5C, D).

Immunofluorescence analysis revealed that, compared to the mCherry + CCI group, macrophage infiltration in the DRG significantly decreased in the hM3D(Gq) + CCI mice (Iba-1^+^) and notably increased in the hM4D(Gi) + CCI group. The overall difference was statistically significant (Fig. 5F, H). Similarly, the proportion of CGRP-positive neurons within the DRG significantly diminished in the hM3D(Gq) + CCI mice and prominently increased in the hM4D(Gi) + CCI group. The aggregate difference was statistically significant (Fig. 5G, I).

These findings suggest that stimulating the BNST-PVN neural circuit can diminish macrophage infiltration into the DRG and reduce the proportion of CGRP-positive neurons within the DRG, thereby mitigating the CCI-induced decline in MWT. In contrast, inhibiting the neural circuit exhibits an opposite trend. However, modulating the BNST-PVN neural circuit does not impact the level of splenic macrophages.

### Inhibition of the BNST-PVN Neural Circuit Augments Macrophage Infiltration into the DRG of CCI Mice, an Effect Reversed by SE

**Fig. 6:**
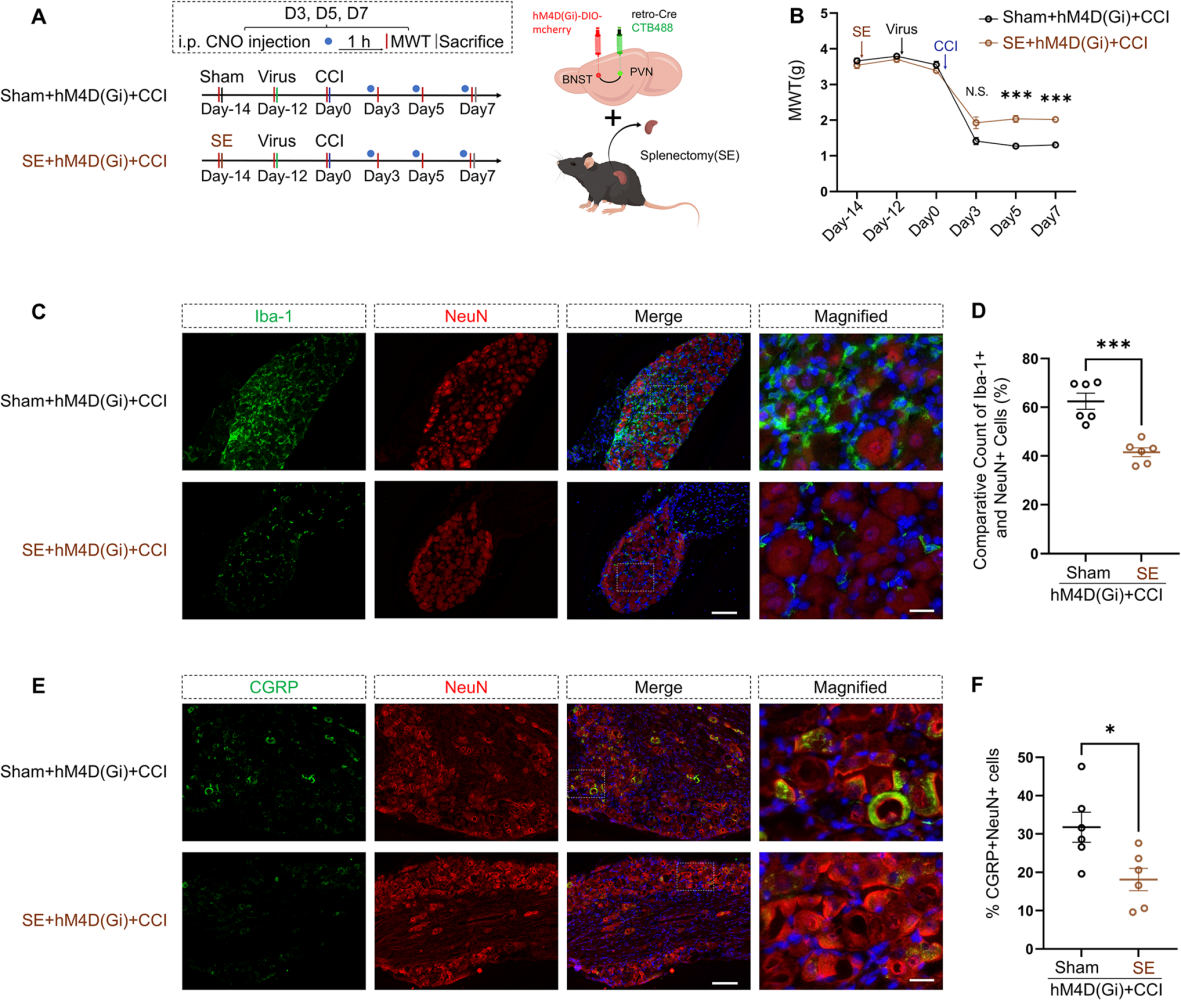
Splenectomy (SE) ameliorates CCI-induced neuropathic pain exacerbated by inhibition of the BNST-PVN neural circuit**. A** Experimental timeline. Schematic of the viral injection sites in the BNST and PVN, along with the splenectomy procedure. **B** The MWT shows significant increases in SE + hM4D(Gi)mice on days 5 and 7 post-CCI surgery compared to Sham + hM4D(Gi) mice (repeated measures two-way ANOVA, time: *F*_(2.980, 29.80)_ = 280.5, *P <* 0.0001; SE: *F*_(1, 10)_ = 32.22, *P =* 0.0002; interaction: *F*_(5, 50)_ = 11.81, *P <* 0.0001; *n* = 6 per group). ***P < 0.001, N.S.: no significant difference. **C, D** Immunofluorescence images of the DRG showing Iba-1^+^ (green) and NeuN^+^ (red) cells. Quantification of Iba-1^+^ cells relative to NeuN^+^ cells in the DRG shows a significant reduction in SE + hM4D(Gi) mice compared to Sham + hM4D(Gi) mice (two-tailed unpaired Student’s *t*-test, *t* = 5.526, *df* = 10, *P =* 0.0003; *n* = 6 per group). ***P < 0.001. Scale bars, 100 µm, magnified 20 µm. **E, F** Immunofluorescence images of the DRG showing CGRP^+^ (green) and NeuN^+^ (red) cells. Quantification of CGRP^+^ cells relative to NeuN^+^ cells in the DRG shows a significant reduction in SE + hM4D(Gi) mice compared to Sham + hM4D(Gi) mice (two-tailed unpaired Student’s *t*-test, *t* = 2.797, *df* = 10, *P =* 0.0189; *n* = 6 per group). *P < 0.05. Scale bars, 100 µm, magnified 20 µm. Data are presented as the mean ± SEM.

Our previous findings suggest that the BNST-PVN neural circuit regulates the neuropathic pain caused by CCI and maintains a direct neural connection with the spleen. We hypothesized that the spleen could play a pivotal role in this process. To investigate this hypothesis, we performed either the SE procedure or sham surgery on mice. After a two-day recovery period, we administered chemical-genetic virus injections and performed CCI procedures on the 14th day following the splenectomies (Fig. 6A).

MWT was assessed 1 h after administering an intraperitoneal injection of CNO. Compared to the Sham + hM4D(Gi) + CCI group, the SE + hM4D(Gi) + CCI mice demonstrated notably enhanced MWT on day 5 (*P =* 0.0007) and day 7 (*P =* 0.0002) post-CCI, with no significant difference on day 3 (*P =* 0.1495). An overall statistically significant difference was found (Fig. 6B). Immunofluorescence staining showed a significant reduction in macrophage infiltration (Iba-1^+^) in the DRG of the SE + hM4D(Gi) + CCI mice (Fig. 6C, D). Concurrently, there was a significant decrease in the proportion of CGRP-positive neurons (Fig. 6E, F).

### SD Mitigates the Increase of Macrophage Infiltration in the DRG Induced by Inhibiting the BNST-PVN Neural Circuit in CCI Mice

**Fig. 7:**
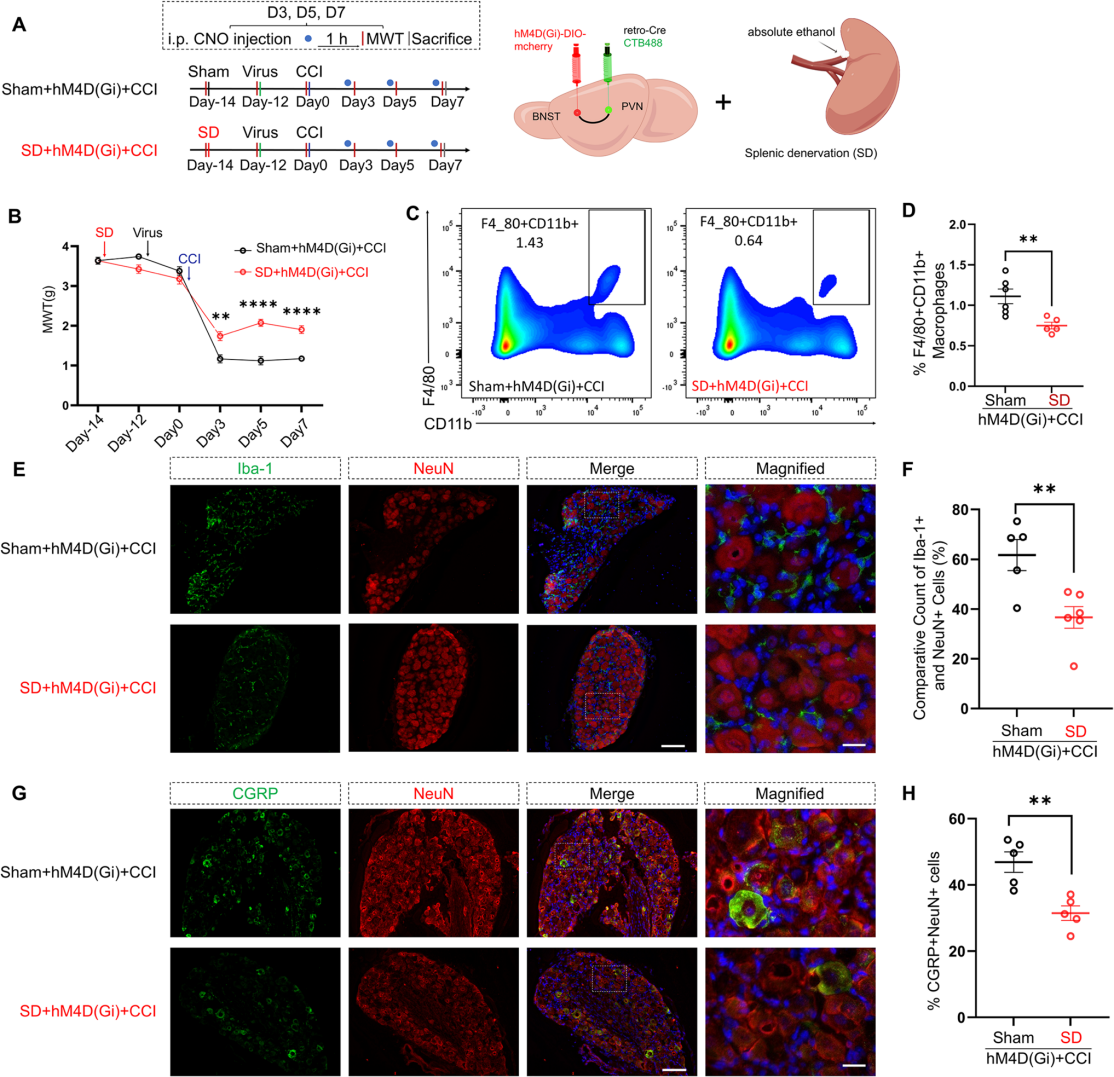
Splenic denervation (SD) mitigates CCI-induced neuropathic pain exacerbated by inhibition of the BNST-PVN neural circuit. **A** Experimental timeline. Schematic of the experimental setup, including viral injections into the BNST and PVN and the SD procedure using absolute ethanol. **B** The MWT measurements over time, showing significant increases in SD + hM4D(Gi) mice on days 3, 5, and 7 post-CCI surgery compared to Sham + hM4D(Gi) mice (repeated measures two-way ANOVA, time: *F*_(3.527, 74.07)_ = 295.9, *P <* 0.0001; SD: *F*_(1, 21)_ = 18.15, *P =* 0.0003; interaction: *F*_(5, 105)_ = 17.68, *P <* 0.0001; Sham, *n* = 12; SD, *n* = 11). **P < 0.01, ****P < 0.0001. **C, D** Representative flow cytometry plots showing the population of F4/80^+^ and CD11b^+^ macrophages in the spleen for Sham + hM4D(Gi) and SD + hM4D(Gi) mice. Quantification of F4/80^+^ and CD11b^+^ macrophages in the spleen, indicating a significant reduction in the SD group compared to Sham (two-tailed unpaired Student’s* t*-test, *t* = 3.334, *df* = 9, *P =* 0.0087; Sham, *n* = 6; SD, *n* = 5). **P < 0.01. **E, F** Immunofluorescence images of the DRG showing Iba-1^+^ (green) and NeuN^+^ (red) cells, with merged and magnified views. Scale bar, 100 µm. Quantification of Iba-1^+^ cells relative to NeuN^+^ cells in the DRG shows a significant reduction in SD + hM4D(Gi) mice compared to Sham + hM4D(Gi) mice (two-tailed unpaired Student’s *t*-test, *t* = 3.377, *df* = 9, *P =* 0.0082; Sham, *n* = 5; SD, *n* = 6). **P < 0.01. Scale bars, 100 µm, magnified 20 µm. **G, H** Immunofluorescence images of the DRG showing CGRP^+^ (green) and NeuN^+^ (red) cells, with merged and magnified views. Scale bar, 100 µm. Quantification of CGRP^+^ cells relative to NeuN^+^ cells in the DRG shows a significant reduction in SD + hM4D(Gi) mice compared to Sham + hM4D(Gi) mice (two-tailed unpaired Student’s *t*-test, *t* = 4.075, *df* = 8, *P =* 0.0036; Sham, *n* = 4; SD, *n* = 6). **P < 0.01. Scale bars, 100 µm, magnified 20 µm. Data are presented as the mean ± SEM.

To build upon our preliminary findings, we performed either an SD procedure or sham surgery on mice. After a two-day recovery period, the mice underwent chemical-genetic virus injections and the CCI procedure on the 14th day following the SD procedure (Fig. 7A).

MWT was assessed 1 h after intraperitoneal injection of CNO. The SD + hM4D(Gi) + CCI group demonstrated significant elevations in MWT on day 3 (*P =* 0.0072), day 5 (*P <* 0.0001), and day 7 (*P <* 0.0001) post-CCI surgery. These results were statistically significant (Fig. 7B). Flow cytometry results revealed a notable decrease in the proportion of macrophages (F4/80^+^ and CD11b^+^ cells) in the spleen of mice post-SD as compared to the control group (Fig. 7C, D). Immunofluorescence inspection disclosed a significant reduction in macrophage infiltration within the DRG of the SD + hM4D(Gi) + CCI mice (Iba-1^+^) (Fig. 7E, F). Moreover, a considerable decline occurred in the proportion of CGRP-positive neurons (Fig. 7G, H).

### SS Mitigates the Modulating Influence of the BNST-PVN Neural Circuit on CCI

**Fig. 8:**
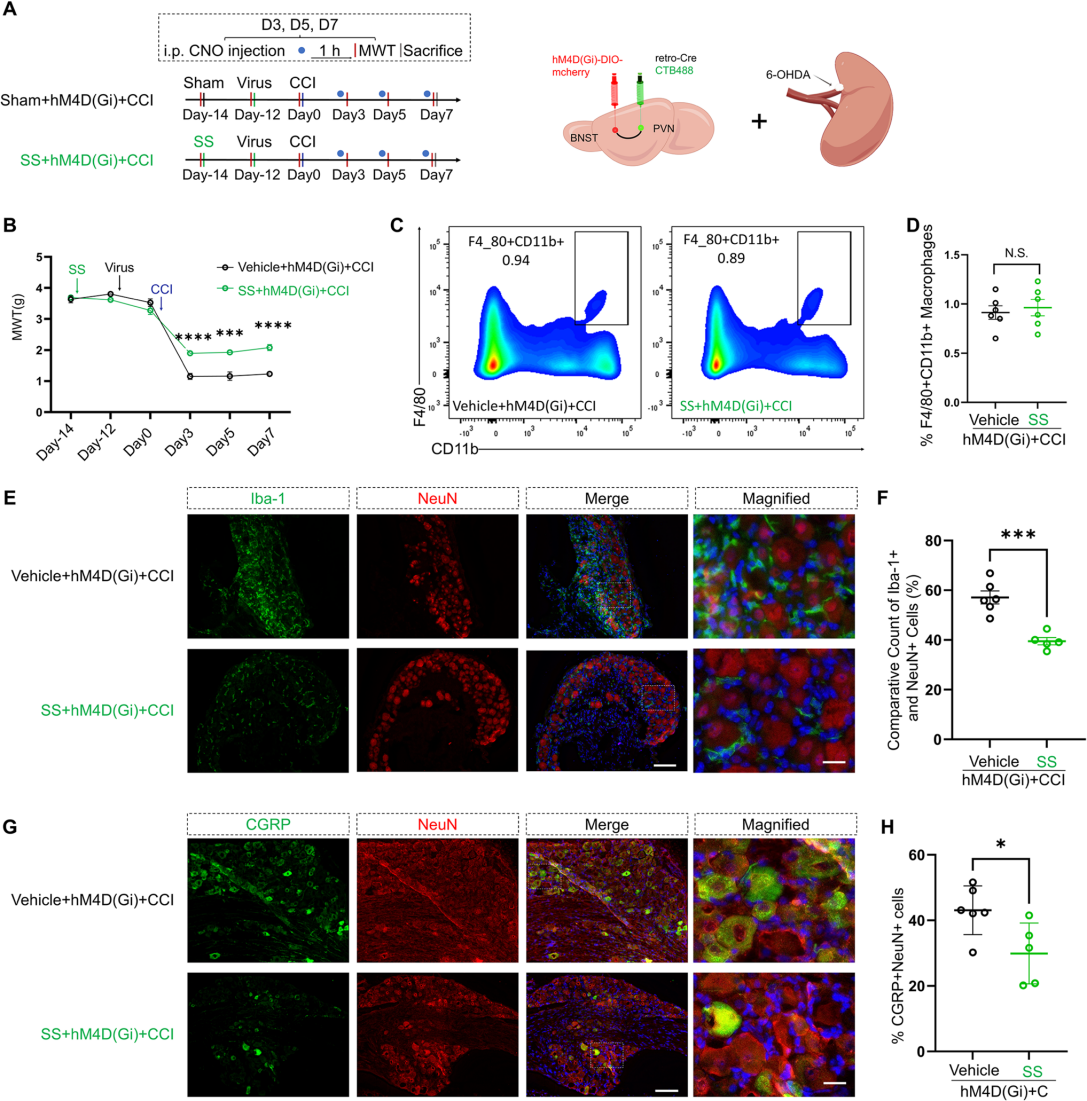
SS mitigates CCI-induced neuropathic pain exacerbated by BNST-PVN neural circuit inhibition. **A** Experimental timeline depicting the procedures undertaken. Schematic illustrating the targeted delivery of viruses to the BNST and PVN and the SS procedure, including the chemical sympathectomy agent 6-OHDA. **B** Graph showing the MWT over time, with significant increases in MWT in SS + hM4D(Gi) mice on days 3, 5, and 7 post-CCI surgery compared to Vehicle + hM4D(Gi) mice, indicating an amelioration of pain (repeated measures two-way ANOVA, time: *F*_(3.397, 74.74)_ = 272.6, *P <* 0.0001; SS: *F*_(1, 22)_ = 31.32, *P <* 0.0001; interaction: *F*_(5, 110)_ = 14.47, *P <* 0.0001; *n* = 12 per group). ***P < 0.001, ****P < 0.0001. **C, D** Flow cytometry analysis of splenic cells. Representative plots display the populations of F4/80^+^ and CD11b^+^ cells. Quantitative analysis of the percentage of F4/80^+^CD11b^+^ cells in the spleen, confirming no significant differences between the groups (two-tailed unpaired Student’s *t*-test, *t* = 0.4626, *df* = 10, *P =* 0.6536; *n* = 6 per group). N.S.: no significant difference. **E, F** Immunofluorescence images of the DRG showing Iba-1^+^ (green) and NeuN^+^ (red) cells. Quantification of Iba-1^+^ cells relative to NeuN^+^ cells in the DRG, with SS + hM4D(Gi) mice showing a reduced count (two-tailed unpaired Student’s *t*-test,* t* = 5.585, *df* = 9, *P =* 0.0003; vehicle, *n* = 6; SS, *n* = 5). ***P < 0.001. Scale bars, 100 µm, magnified 20 µm. **G, H** Immunofluorescence images of the DRG showing CGRP^+^ (green) and NeuN^+^ (red) cells, with merged and magnified views. Quantification of the percentage of CGRP^+^ cells relative to NeuN^+^ cells in the DRG. SS + hM4D(Gi) mice show a lower ratio, indicating reduced CGRP expression compared to the vehicle group (two-tailed unpaired Student’s* t*-test, *t* = 2.622, *df* = 9, *P =* 0.0277; vehicle, *n* = 6; SS, *n* = 5). *P < 0.05. Scale bars, 100 µm, magnified 20 µm. Data are presented as the mean ± SEM.

Building upon these insights, we hypothesized that the BNST-PVN neural circuit modulates CCI-induced pain through the sympathetic nerves of the spleen. We performed either an SS procedure or sham surgery. After a two-day recovery period, the mice were subjected to chemical-genetic virus injections and a CCI procedure on the 14th day following the SS procedure (Fig. 8A).

MWT was assessed 1 h after an intraperitoneal injection of CNO. The MWT significantly increased on day 3 (*P <* 0.0001), day 5 (*P =* 0.0007), and day 7 (*P <* 0.0001) following the CCI surgery in the mice that underwent SS. The differences were statistically significant (Fig. 8B). Flow cytometry analysis of the spleen revealed no significant differences in the proportion of macrophages (F4/80^+^ and CD11b^+^ cells) between the SS + hM4D(Gi) + CCI group and the vehicle + hM4D(Gi) + CCI group (Fig. 8C, D). Immunofluorescence staining of the DRG showed a significant reduction in macrophage infiltration in the SS + hM4D(Gi) + CCI mice (Iba-1^+^) (Fig. 8E, F), and the ratio of M1 phenotype (CD86^+^) to macrophages (Iba-1^+^) in the DRG was also reduced (Fig. S3C, D). Concurrently, there was a significant decline in the proportion of CGRP-positive neurons (Fig. 8G, H).

## Discussion

The major findings of this study are as follows: First, interventions such as SE, SD, or SS significantly increased MWT, which indicates a decrease in mechanical allodynia, and they also reduced macrophage infiltration in the DRG of CCI mice. These findings indicate that the spleen contributes to neuropathic pain modulation in CCI mice. Second, PRV injections into the spleen identified direct neural connections to the BNST and PVN in the brain, but not to other visceral organs, reinforcing the existence of a specific spleen-brain axis. Third, chemogenetic manipulation of the BNST-PVN circuit revealed that inhibiting this pathway increased macrophage infiltration in the DRG and increased mechanical allodynia. These effects were reversed by SE, SD, or SS, underscoring the pivotal role of the spleen-regulated BNST-PVN circuit in neuropathic pain, suggesting it as a potential therapeutic target.

The spleen, an essential immune organ, has been increasingly recognized for its role in modulating neuropathic pain. Recent studies have shown that the spleen can influence pain through its interaction with neural circuits and immune responses. For instance, Zhang and colleagues [30] demonstrated that neurons in the CeA and the PVN that express corticotropin-releasing hormone (CRH) are connected to the splenic nerve. Ablation or chemogenetic inhibition of these neurons reduces plasma cell formation, whereas chemogenetic activation increases plasma cell abundance after immunization. This study highlights the brain’s control of adaptive immunity and suggests the possibility of enhancing immunocompetency through behavioral intervention [30].

In our study, we found that interventions such as SE, SD, or SS significantly decreased mechanical allodynia and reduced macrophage infiltration in the DRG of CCI mice. These findings suggest that the spleen’s immune function plays a crucial role in modulating neuropathic pain. Previous studies have demonstrated that spinal microglia are critical in the development of neuropathic pain [32]. In addition, macrophages secrete inflammatory mediators that upregulate the expression of nociceptive peptides such as CGRP in sensory neurons [33, 34]. Although our study primarily focused on quantifying macrophage numbers in the DRG, our findings indicate that macrophage infiltration is correlated with CGRP upregulation. In our study, “infiltration” refers to the net accumulation of macrophages in the DRG, which can result from both migration (recruitment from circulation) and local proliferation. Both SD and SS produce anti-inflammatory effects by disrupting sympathetic nervous system activity within the spleen [35–37]. A recent study demonstrated that SD attenuates the production of tumor necrosis factor in the spleen and serum of mice with endotoxemia [38]. This disruption by SD or SS leads to a decrease in pro-inflammatory cytokines and mediators, modulation of immune cell activity, and consequently, a reduction in both local and systemic inflammation. Furthermore, our experiments revealed that SD or SS significantly ameliorated neuropathic pain in CCI mice through these anti-inflammatory mechanisms. Collectively, these findings highlight the therapeutic benefits of targeting splenic sympathetic innervation in inflammatory and autoimmune conditions.

Direct injection of PRV into the spleen retrogradely labeled neurons in brain regions such as the BNST and PVN, but not in other visceral organs, including the liver, lung, heart, kidney, and colon. This strongly suggests a direct connection between the spleen and specific brain regions. Chemogenetic manipulation of the BNST-PVN circuit showed that inhibiting this pathway increased macrophage infiltration in the DRG and increased mechanical allodynia. Interestingly, these effects were reversed by interventions such as SE, SD, or SS. Recent research has shown that activation of glutamatergic projections or inhibition of GABAergic projections from the anteroventral BNST to PVN CRH neurons regulates maternal separation-induced visceral pain [31, 39]. Another study has demonstrated that the lateral hypothalamus-projecting BNST plays a role in chronic pain-induced anxiety-like behaviors [40]. Using the DREADD system, we found that the BNST-PVN neural circuit plays a role in neuropathic pain in CCI mice. Collectively, it seems that both the BNST and PVN play crucial roles in pain modulation.

The PVN serves as a critical autonomic control center that modulates sympathetic outflow. While the PVN is best known for its role in the hypothalamic-pituitary-adrenal axis, it also exerts direct descending control over sympathetic preganglionic neurons located in the intermediolateral cell column of the spinal cord. These preganglionic neurons project to the celiac and superior mesenteric ganglia, which in turn provide sympathetic innervation to the spleen. The spleen receives sympathetic fibers from the celiac plexus (and adjacent plexuses), originating from spinal segments roughly T6–T10 under hypothalamic control. Catecholamine release and other sympathetic signals in the spleen can influence the mobilization and phenotype of immune cells residing in or transiting through the splenic compartment [41, 42]. In addition, sympathetic signaling can regulate both the release of monocytes/macrophages from the spleen and their functional activation state [43]. Collectively, this suggests that the PVN regulates splenic sympathetic innervation, which in turn influences macrophage numbers and modulates pain.

A recent study has revealed that splenic T helper 2 (Th2) immune responses in male mice are differentially regulated in acute and chronic neuropathic pain *via* distinct neural pathways involving the dorsal motor nucleus of the vagus (ACh^DMV^) [13]. In acute pain, there is increased excitation from glutamatergic neurons in the primary somatosensory cortex to ACh^DMV^ neurons, which enhances splenic Th2 cells. Conversely, chronic pain results in increased inhibition from GABAergic neurons in the amygdala to ACh^DMV^ neurons, leading to a reduction in splenic Th2 cells. This illustrates how the brain encodes immune responses in the spleen based on the pain state [13]. Collectively, the spleen plays a crucial role in both acute and chronic pain.

Depression frequently occurs alongside neuropathic pain, creating a complex and challenging clinical scenario [44–46]. Accumulating evidence suggests that the spleen plays a significant role in modulating depression-like behaviors through its involvement in immune responses, inflammation, and neuroimmune interactions [14–16]. Many rodent studies have shown that lipopolysaccharide (LPS)-induced systemic inflammation is associated with splenomegaly and depression-like behaviors [18, 21, 47]. Research has shown that SD significantly blocks systemic inflammation and depression-like behavior, alters the composition of gut microbiota in LPS-treated mice [18], and blocks depression-like behaviors in *Chrna7-*KO mice [48]. A recent study has shown that SE blocks the antidepressant-like effects of the new antidepressant arketamine in a chronic social defeat stress model [24]. Given the crucial role of the spleen in immune responses, cytokine production, and neuroimmune interactions, it is likely that the spleen plays a critical role in neuropathic pain and its associated depression. Therefore, targeting splenic activity and cytokine production through anti-inflammatory treatments may help alleviate pain and depression.

Despite the significant findings of our study, several limitations must be acknowledged. First, although we demonstrated the immunomodulatory effects of splenic interventions on DRG macrophages in the CCI model, we cannot definitively confirm that the infiltrating cells in the DRG originated directly from the spleen. *In vivo* fluorescence imaging techniques could potentially track these cells; however, such methods are technically challenging due to the small size of the DRG and its anatomical location within surrounding bone structures. Second, our study primarily relied on immunofluorescence to detect macrophages in the DRG. While flow cytometry might offer a more comprehensive analysis of immune cell counts and phenotypes, the limited tissue volume of the DRG and its dense neural fiber network make it difficult to obtain sufficient cells for this method. In addition, current *in vivo* digestion techniques for DRG tissue are not well-established. These technical limitations highlight the need to develop more advanced methods for studying immune cell trafficking and characterization in small neural tissues like the DRG.

In conclusion, our study underscores the critical role of the spleen in modulating neuropathic pain through the BNST-PVN neural circuit. Interventions such as SE, SD, or SS significantly decreased mechanical allodynia and reduced macrophage infiltration in the DRG of CCI mice. In addition, the spleen's neural connections with the brain, particularly the BNST and PVN, highlight its influence on neuropathic pain. Chemogenetic manipulation of the BNST-PVN circuit demonstrated that inhibiting this pathway increased macrophage infiltration in the DRG and increased mechanical allodynia, effects reversed by SE, SD, or SS. These findings suggest that the spleen, through its immune and neural functions, plays a pivotal role in the modulation of neuropathic pain.

## Supplementary Information

Below is the link to the electronic supplementary material.

Supplementary file1 (PDF 689 KB)
