# Supplementary material for: The Bed Nucleus of the Stria Terminalis—Paraventricular Nucleus of the Hypothalamus Neural Circuit Regulates Neuropathic Pain Through the Brain-Spleen Axis
Source: Neurosci Bull. 2025 Aug 20;41(12):2148–66. doi: 10.1007/s12264-025-01454-9 (PMC12698908; doi:10.1007/s12264-025-01454-9)
Supplement: Supplementary file 1 — Supplementary file1 (PDF 689 KB) [file 12264_2025_1454_MOESM1_ESM.pdf]

Supplementary Materials

Supplementary Figures

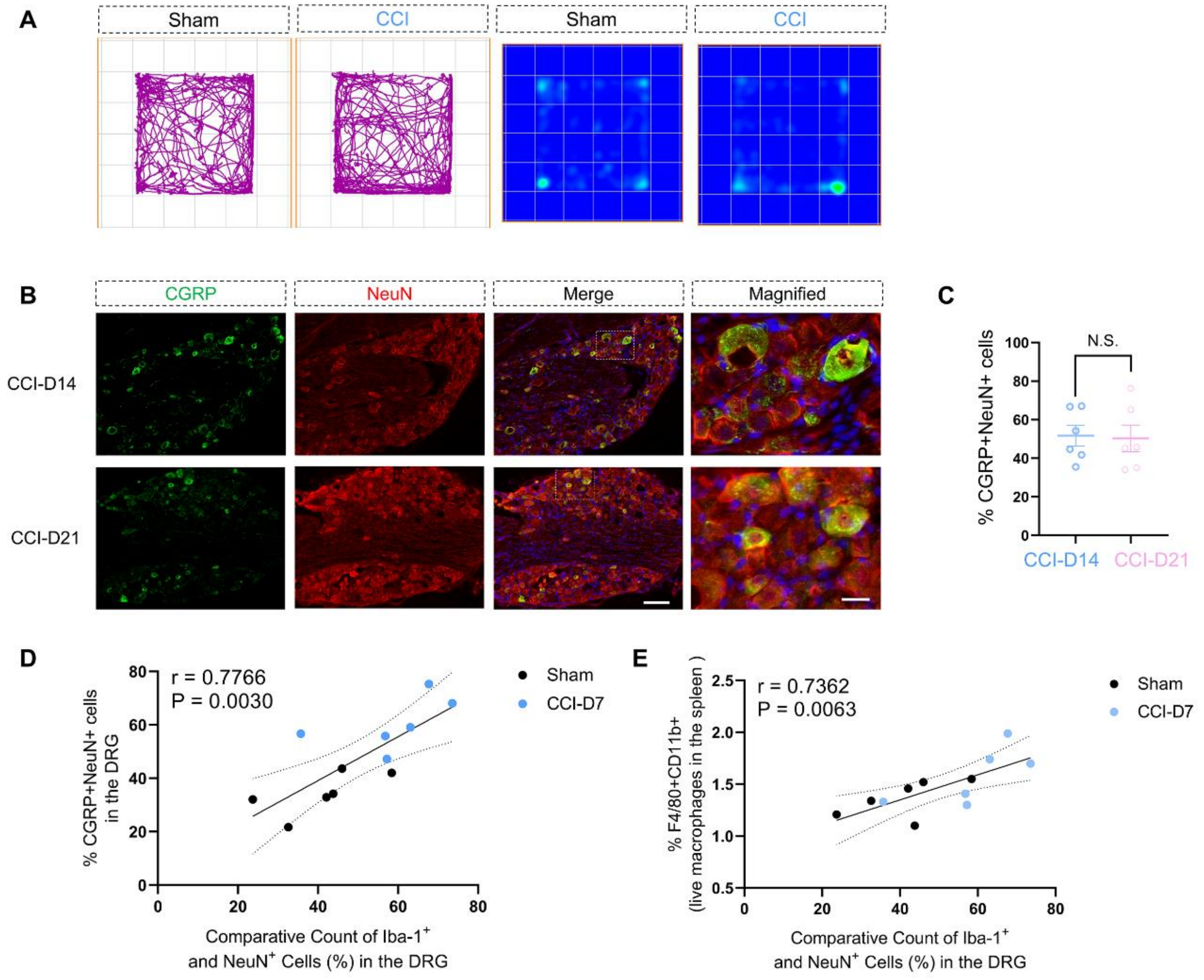

Fig. S1. Locomotor activity and immunofluorescence in the DRG of CCI mice. **A** Representative images of the open field test (OFT) showing the movement paths of Sham and CCI mice. The left panel shows the total distance traveled, and the right panel shows the heatmap of movement intensity. **B-C** Immunofluorescence images of the DRG showing $CGRP^+$ (green) and $NeuN^+$ (red) cells at 14 and 21 days post-CCI. The merged images and magnified views indicate the distribution and co-localization of $CGRP^+$ and $NeuN^+$ cells. Quantification of the percentage of $CGRP^+$ cells relative to $NeuN^+$ cells in the DRG on days 14 and 21 post-CCI, showing no significant difference between the

two time points (two-tailed unpaired Student's t-test, t = 0.1592, df = 10, P = 0.8766; n = 6 per group). N.S.: not significant. Scale bar: 100 μm (Magnified 20μm). **D** There was a positive correlation (r = 0.7766, P = 0.0030) between $CGRP^{+}NeuN^{+}$ cells / $NeuN^{+}$ cells and $Iba1^{+}$ cells / $NeuN^{+}$ cells in the DRG. **E** There was a positive correlation (r = 0.7362, P = 0.0063) between $F4/80^{+}CD11b^{+}$ cells in the spleen and $Iba1^{+}$ cells / $NeuN^{+}$ cells in the DRG. Data are presented as mean ± SEM.

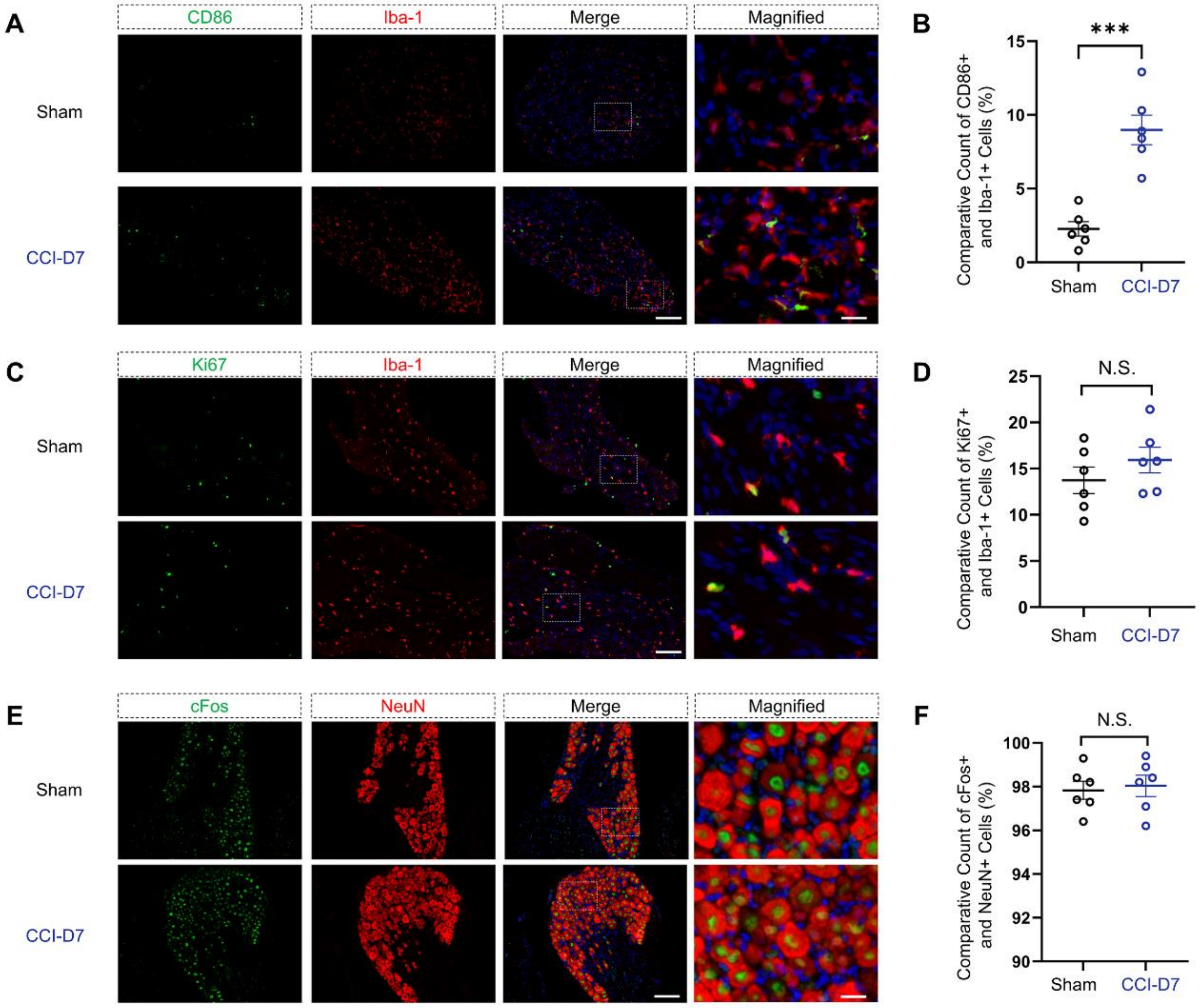

Fig. S2. Immunofluorescence in the DRG of CCI mice. **A-B** Immunofluorescence images of the dorsal root ganglion (DRG) showing $CD86^{+}$ (green) and $Iba1^{+}$ (red) cells. Quantification of $CD86^{+}$ cells relative to $Iba\text{-}1^{+}$ cells in the DRG, with CCI mice showing a higher comparative count (two-tailed unpaired Student's t-test, t = 6.059, df = 10, P = 0.0001; n = 6 per group). ***P < 0.001. Scale bar:

100 μm (Magnified 20μm). **C-D** Immunofluorescence images of the dorsal root ganglion (DRG) showing Ki67$^+$ (green) and Iba1$^+$ (red) cells. Quantification of Ki67$^+$ cells relative to Iba-1$^+$ cells in the DRG, with CCI mice showing no statistically significant difference (two-tailed unpaired Student's t-test, t =1.094, df = 10, P = 0.2997; n = 6 per group). N.S.: not significant. Scale bar: 100 μm (Magnified 20μm). **E-F** Immunofluorescence images of the dorsal root ganglion (DRG) showing cFos$^+$ (green) and NeuN$^+$ (red) cells. Quantification of cFos$^+$ cells relative to NeuN$^+$ cells in the DRG, with CCI mice showing no statistically significant difference (two-tailed unpaired Student's t-test, t =0.3143, df = 10, P = 0.7597; n = 6 per group). N.S.: not significant. Scale bar: 100 μm (Magnified 20μm). Data are presented as mean ± SEM.

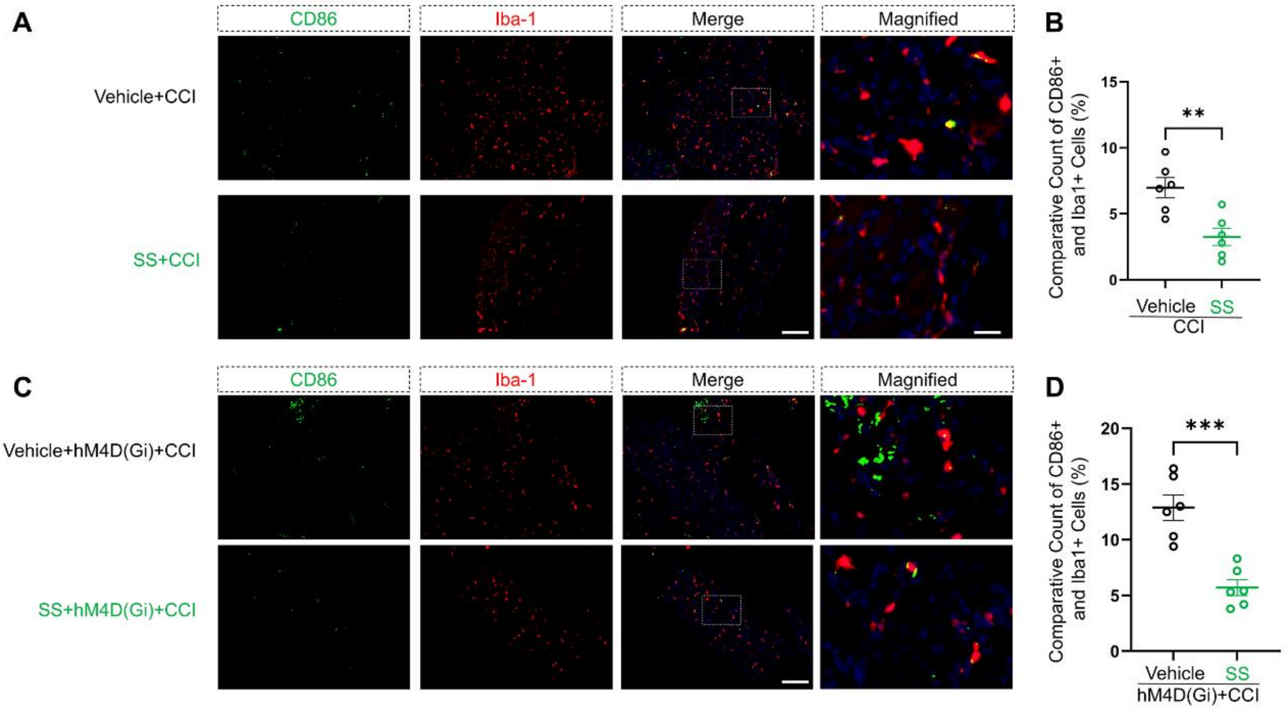

Fig. S3. Immunofluorescence in the DRG of SS + CCI or SS + hM4D(Gi) + CCI mice**. A-B** Immunofluorescence images of the dorsal root ganglion (DRG) showing CD86$^+$ (green) and Iba1$^+$ (red) cells. Quantification of CD86$^+$ cells relative to Iba-1$^+$ cells in the DRG, with SS + CCI mice showing a lower comparative count (two-tailed unpaired Student's t-test, t = 3.733, df = 10, P = 0.0039; n = 6

per group). **$P < 0.01$. Scale bar: 100 μm (Magnified 20μm). **C-D** Immunofluorescence images of the dorsal root ganglion (DRG) showing $CD86^+$ (green) and $Iba1^+$ (red) cells. Quantification of $CD86^+$ cells relative to Iba-1^+ cells in the DRG, with SS + hM4D(Gi) + CCI mice showing a lower comparative count (two-tailed unpaired Student's t-test, $t = 5.335$, $df = 10$, $P = 0.0003$; $n = 6$ per group). ***$P < 0.001$. Scale bar: 100 μm (Magnified 20μm). Data are presented as mean ± SEM.

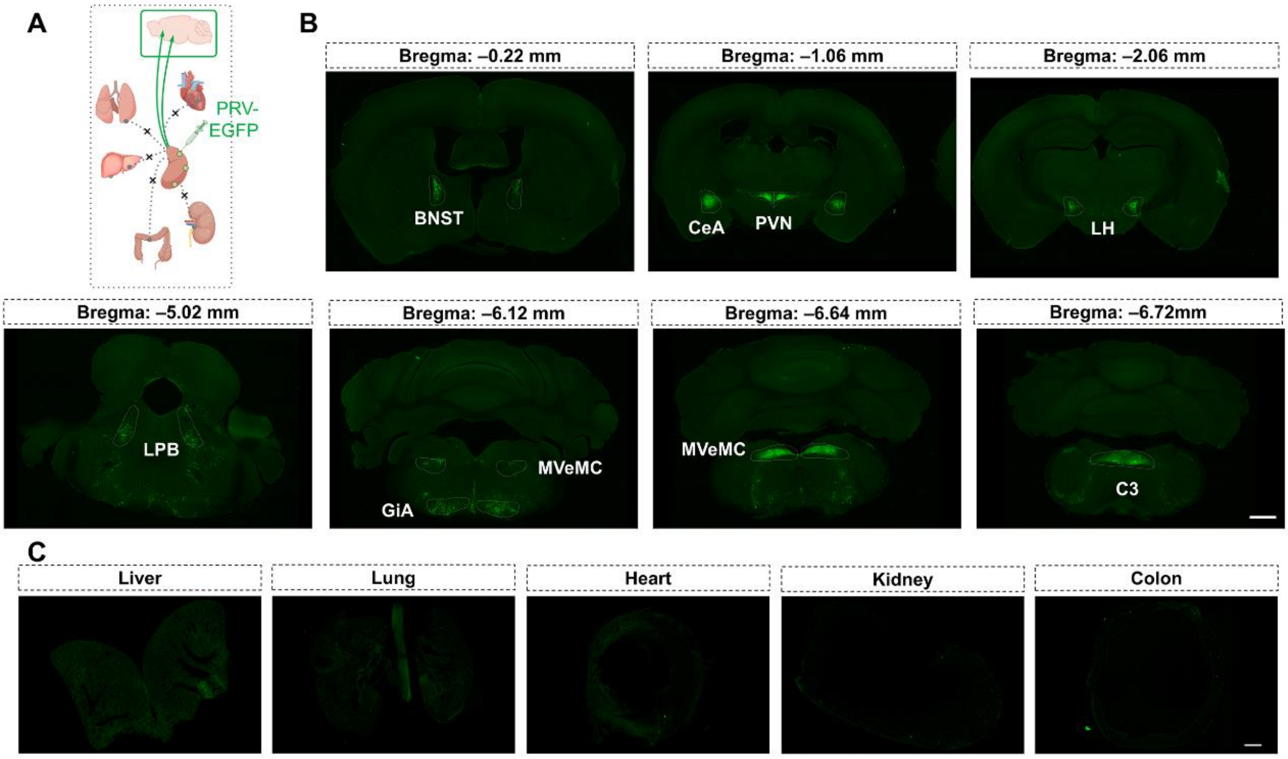

Fig. S4. BNST and PVN are retrogradely traced by spleen injection of PRV-EGFP. **A** Schematic representation of PRV-EGFP injection into the spleen to trace neural connections retrogradely. **B** Brain sections collected five days post-PRV injection show neurons labeled with green fluorescence in various brain regions, including the bed nucleus of the stria terminalis (BNST), central amygdala (CeA), paraventricular nucleus (PVN), lateral hypothalamus (LH), medial vestibular nucleus (MVeMC), Lateral parabrachial nucleus (LPB), gigantocellular nucleus (GiA), and adrenergic cell group C3 (C3). The sections are shown at different Bregma levels: -0.22 mm, -1.06 mm, -2.06 mm, -5.02 mm, -6.64 mm, and -6.72 mm. Scale bar: 500 μm. **C** No green fluorescence-labeled neurons were

observed in the heart, liver, lungs, kidneys, or colon sections, confirming the specificity of PRV-EGFP tracing to the spleen-brain axis. Scale bar: 500 μm.
